# Tumors overcome the action of the wasting factor ImpL2 by locally elevating Wnt/Wingless

**DOI:** 10.1073/pnas.2020120118

**Published:** 2021-06-02

**Authors:** Jiae Lee, Katelyn G.-L. Ng, Kenneth M. Dombek, Dae Seok Eom, Young V. Kwon

**Affiliations:** ^a^Department of Biochemistry, School of Medicine, University of Washington, Seattle, WA 98195;; ^b^Department of Developmental and Cell Biology, School of Biological Sciences, University of California, Irvine, CA 92697

**Keywords:** cachexia, tumor, Yorkie, ImpL2, Wnt

## Abstract

Tumors often secrete wasting factors associated with atrophy and the degeneration of host tissues. If tumors were to be affected by the wasting factors, mechanisms allowing tumors to evade the adverse effects of the wasting factors must exist, and impairing such mechanisms may attenuate tumors. We use *Drosophila* midgut tumor models to show that tumors up-regulate Wingless (Wg) to oppose the growth-impeding effects caused by the wasting factor, ImpL2 (insulin-like growth factor binding protein [IGFBP]-related protein). Growth of Yorkie (Yki)-induced tumors is dependent on Wg while either elimination of *ImpL2* or elevation of insulin/insulin-like growth factor signaling in tumors revokes this dependency. Notably, Wg augmentation could be a general mechanism for supporting the growth of tumors with elevated ImpL2 and exploited to attenuate muscle degeneration during wasting. Our study elucidates the mechanism by which tumors negate the action of ImpL2 to uphold their growth during cachexia-like wasting and implies that targeting the Wnt/Wg pathway might be an efficient treatment strategy for cancers with elevated IGFBPs.

## Significance

The cancer-derived cachectic factors induce wasting in the patient’s tissues, including muscle and adipose tissue. If cancers were to be equally affected by the cachectic factors, they must be wasted away. Nevertheless, cancers appear to grow during cachexia, suggesting a mechanism for protecting themselves from the cachectic factors. We employ *Drosophila* tumor models to demonstrate that a signaling protein Wingless works locally to protect tumors from the action of the secreted wasting factor ImpL2. Furthermore, we show that Wingless augmentation specifically in muscle could be exploited to attenuate systemic tissue wasting induced by localized tumors. Altogether, our study provides insights into the mechanism by which tumors evade the adverse effects induced by the wasting factors to avoid self-wasting.

The reduction of tissue mass is a hallmark of the tissue wasting associated with cancers and other chronic conditions (1–4). Notably, a key feature of muscle wasting is catabolism of muscle proteins, which results in muscle mass loss (2, 4). The reduction in tissue mass is also observed in *Drosophila* models of cachexia-like wasting (5–9). Formation of tumors in the midgut by expression of an active form of the transcription factor in the Hippo pathway, Yorkie (Yki^3S/A^) (10), or transplantation of malignant disk tumors into adult flies induces atrophy of ovaries and fat body (5, 6). These tumors express a high level of the secreted *Drosophila* insulin-like peptides (Dilps) antagonist ImpL2, which causes tissue atrophy by reducing systemic insulin/insulin-like growth factor (IGF) signaling (5–7). Similarly, a decrease in IGF1 signaling in wasting muscle is well documented in mammals (11–13). Diminishing Akt activity in the muscle leads to activation of the forkhead transcription factor (FoxO) and autophagy-related 1 (Atg1), which in turn increases protein catabolism (2, 12, 14, 15). Multiple secreted factors contributing to tissue wasting during cachexia have been identified (2, 3, 16). In particular, the transforming growth factor β family members, activins, induce muscle protein catabolism in part by inhibiting Akt signaling (17–19). Given that the growth of cancers accompanies an increase in mass via activation of various anabolic processes, the organismal state under cachexia is expected to be unfavorable for cancer growth; however, it has been shown that cancers grow in a variety of cancer cachexia models (19–22). Conceptually, if cancers were to respond to the secreted wasting factors, these factors would oppose cancer growth. Thus, cancers must have a mechanism to overcome the adverse effects induced by the wasting factors to ensure their growth during cachexia. Nevertheless, it is unclear whether these wasting factors could oppose cancer growth during cachexia and how cancers evade the potentially growth-impeding effects induced by the wasting factors to uphold their growth.

In *Drosophila*, binding of Dilps to insulin-like receptor (InR) initiates the insulin/IGF pathway by turning on phosphoinositide 3-kinases (PI3K), which leads to activation of Akt (Akt1 in *Drosophila*) (23). Akt1 activation promotes growth by inhibiting the *Drosophila* forkhead transcription factor Foxo, which is a growth suppressor, and activating the target of rapamycin (Tor), which is a conserved regulator of cell size and organ growth (23). In turn, Tor inhibits Thor (4E-BP in humans) to enhance translational initiation and activates ribosomal protein S6 kinase (S6k) to increase ribosome biogenesis (23). Additionally, Tor suppresses autophagy by inhibiting Atg1 (24). Thus, maintaining insulin/IGF signaling is crucial for supporting the growth of tissues as well as an organism. In contrast, attenuation of insulin/IGF signaling is associated with tissue wasting in *Drosophila* (5, 6, 25). Recent studies have demonstrated that ImpL2 is a tumor-derived wasting factor, which induces a reduction in systemic insulin/IGF signaling (5, 6). One puzzling observation is that Yki^3S/A^-induced midgut tumors (*yki*^*3S/A*^ tumors) and transplanted malignant disk tumors can grow regardless of the dramatic increase in ImpL2 expression in these tumors. Considering the fundamental role of insulin/IGF signaling in growth, these tumors must have a mechanism to evade the growth-impeding effect induced by ImpL2. It is not known how these tumors maintain insulin/IGF signaling even though ImpL2 is highly up-regulated in these tumors.

In this study, we employ *Drosophila* midgut tumor models to address whether tumors are also subjected to the ramifications of ImpL2 elevation and how tumors overcome the ImpL2-induced adverse effect on their growth. Our results indicate that Wg up-regulation is the mechanism by which *yki*^*3S/A*^ tumors evade the action of ImpL2. Thus, without Wg, *yki*^*3S/A*^ tumor growth is significantly affected by ImpL2. Finally, we show that the Wg-mediated antagonism of the ImpL2 action could be a general principle for supporting the growth of a subtype of midgut tumors with elevated ImpL2 expression and be exploited to alleviate muscle degeneration during wasting.

## Results

### Wg Is Essential for the Growth of *yki*^*3S/A*^ Tumors Only in the Presence of ImpL2.

**Fig. 1.**
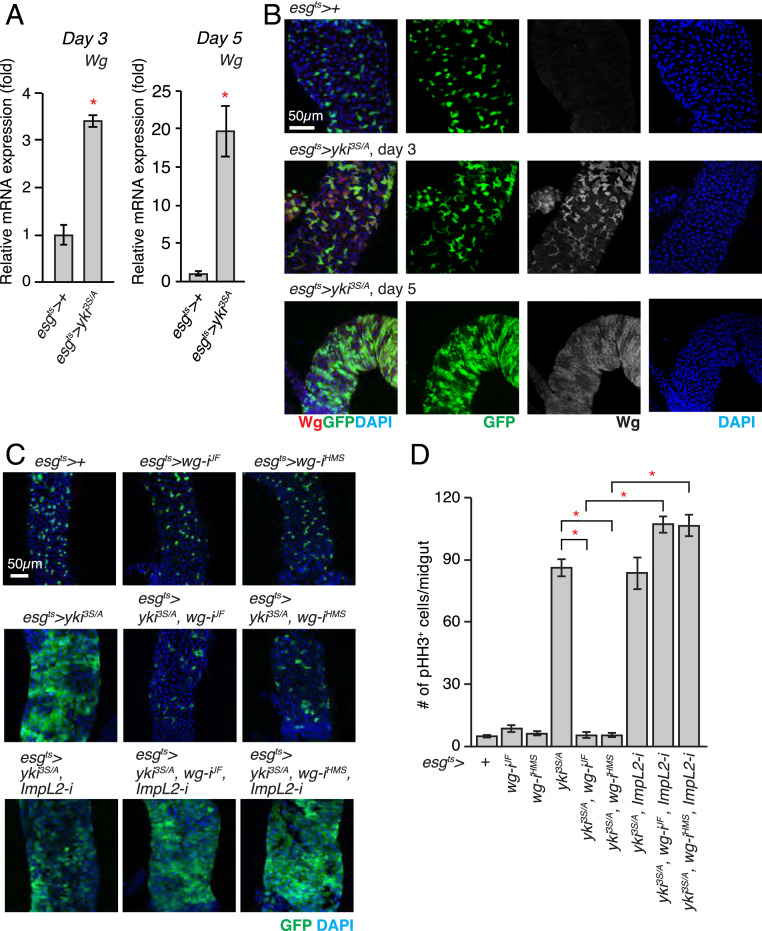
Wg is indispensable for the growth of the *yki*^*3S/A*^ tumor in the presence of *ImpL2*. (*A*) Expression of *wg* mRNA in midguts. The relative abundance of the *wg* transcript in *esg*^*ts*^*>+* or *esg*^*ts*^ *> yki*^*3S/A*^ midguts was determined by qRT-PCR after 3 and 5 d of transgene expression. (*B*) Immunostaining of Wg in posterior midguts. Transgenes were induced for 3 and 5 d. The cells manipulated by *esg*^*ts*^ are marked by GFP (green), Wg staining is shown in red, and nuclei are stained with DAPI (blue) in merged images. (Scale bar, 50 µm.) (*C*) Representative images of posterior midguts. Transgenes were expressed for 5 d. (*D*) Quantification of pHH3^+^ cells per midgut. RNAi lines: *wg-i*^*JF*^, *JF01257*; *wg-i*^*HMS*^, *HMS00794*; *ImpL2-i*, *15009R-3*. *n* = 20 (*esg*^*ts*^*>+*), *n* = 11 (*esg*^*ts*^ *> wg-i*^*JF*^), *n* = 11 (*esg*^*ts*^ *> wg-i*^*HMS*^), *n* = 22 (*esg*^*ts*^ *> yki*^*3S/A*^), *n* = 12 (e*sg*^*ts*^ *> yki*^*3S/A*^*, wg-i*^*JF*^), *n* = 10 (*esg*^*ts*^ *> yki*^*3S/A*^*, wg-i*^*HMS*^), *n* = 12 (*esg*^*ts*^ *> yki*^*3S/A*^*, wg-i*^*JF*^*, ImpL2-i*), *n* = 12 (*esg*^*ts*^ *> yki*^*3S/A*^*, wg-i*^*HMS*^*, ImpL2-i*), *n* = 22 (*esg*^*ts*^ *> yki*^*3S/A*^*, ImpL2-i*) biological replicates. Mean ± SEMs are shown. *P < 0.01, two-tailed unpaired Student’s *t* test compared with control (*esg*^*ts*^*>+*) unless indicated by bracket. See also *SI Appendix*, Figs. S1–S3.

To address the role of Wg signaling in the growth of *yki*^*3S/A*^ tumors, we first assessed whether Wg was expressed in tumors which were generated by expressing *yki*^*3S/A*^ with *esg-GAL4*, *UAS-Green Fluorescent Protein *(*GFP*), *tub-GAL80*^*ts*^ (referred to as *esg*^*ts*^, hereafter; see *Materials and Methods*). Previous studies have demonstrated that Wg is expressed in the visceral muscle and the intestinal epithelial compartment (26, 27). Wg expressed from the visceral muscles is essential for homeostatic intestinal stem cell (ISC) self-renewal (27). In contrast, Wg expressed in enteroblasts (EBs) during tissue damage plays a crucial role in epithelial regeneration (26). Comparison of *wg* messenger RNA (mRNA) levels in control and *yki*^*3S/A*^ tumor midguts revealed that *wg* mRNA expression was significantly elevated in *yki*^*3S/A*^ tumors (Fig. 1*A*). To discern which compartment of *yki*^*3S/A*^ tumor midguts expressed Wg, we stained the midguts with an anti-Wg antibody. Wg signals were significantly elevated in *esg*^*+*^ cells upon expression of *yki*^*3S/A*^ with *esg*^*ts*^ (Fig. 1*B*). In contrast, Wg signals in the visceral muscle remained unchanged in *yki*^*3S/A*^ tumor midguts (*SI Appendix*, Fig. S1). Altogether, these results indicate that Wg is elevated in *yki*^*3S/A*^ cells.

To test the role of Wg in *yki*^*3S/A*^ tumor growth, we depleted *wg* in *yki*^*3S/A*^ cells using two independent *wg* RNA interference (RNAi) lines: *JF01257* and *HMS00794* (28, 29). We confirmed that both *JF01257* and *HMS00794* could effectively reduce Wg levels when they are expressed in *yki*^*3S/A*^ cells (*SI Appendix*, Fig. S2 *A* and *B*). Knockdown of *wg* in ISCs and EBs had no effect on ISC division (Fig. 1 *C* and *D*). In contrast, expression of *wg* RNAi in *yki*^*3S/A*^ tumors with *esg*^*ts*^ significantly reduced cell proliferation, resulting in a complete suppression of tumor growth as well as a few wasting phenotypes (Fig. 1 *C* and *D* and *SI Appendix*, Fig. S2 *C* and *D*). The complete suppression of tumor growth should account for the rescue of the wasting phenotypes caused by *wg* depletion in *yki*^*3S/A*^ cells. *Drosophila* TCF (dTCF, also known as pangolin) is the key transcription factor in the Wg signaling pathway.

To address if Wg signaling in *yki*^*3S/A*^ cells is important for supporting *yki*^*3S/A*^ tumor growth, we expressed a dominant-negative form of dTCF (dTCF^DN^) with *esg*^*ts*^ to perturb Wg signaling in a cell-autonomous manner. We found that expression of dTCF^DN^ in *yki*^*3S/A*^ cells was sufficient to suppress *yki*^*3S/A*^ tumor growth (*SI Appendix*, Fig. S3*A*), indicating that Wg signaling in *yki*^*3S/A*^ cells plays a key role in *yki*^*3S/A*^ tumor growth. In contrast, expression of dTCF^DN^ in the neighboring enterocytes (ECs) did not significantly alter *yki*^*3S/A*^ tumor growth (*SI Appendix*, Fig. S3*B*), suggesting that Wg signaling in ECs does not contribute to *yki*^*3S/A*^ tumor growth if there is any Wg signaling in them.

ImpL2 antagonizes Dilps, which leads to a reduction in insulin/IGF signaling, with an exception in a small subset of neurons in the larval brain (30–35). Given the suggested role of Wg in promoting the progression of eye disk tumors under insulin resistance induced by a high-sugar diet (36), we hypothesized that *yki*^*3S/A*^ tumor-derived Wg might negate the adverse effect caused by ImpL2 elevation to support tumor growth. If this is correct, the tumor-growth defect caused by *wg* depletion should be rescued by *ImpL2* depletion in *yki*^*3S/A*^ tumors. Previously, we showed that *ImpL2* was dispensable for the growth of *yki*^*3S/A*^ tumors (5). Consistently, *ImpL2* depletion did not alter the proliferation of *yki*^*3S/A*^ cells (Fig. 1*C*). Of significance, expression of *ImpL2* RNAi with *esg*^*ts*^ completely rescued the defect in *yki*^*3S/A*^ cell proliferation caused by *wg* knockdown, leading to the formation of fully grown tumors (Fig. 1 *C* and *D*). Altogether, these results demonstrate that Wg is crucial for the growth of *yki*^*3S/A*^ tumors only when *ImpL2* is present.

### Augmentation of Insulin-Akt Signaling Is Sufficient to Rescue the Tumor-Growth Defect Caused by *wg* Depletion.

**Fig. 2.**
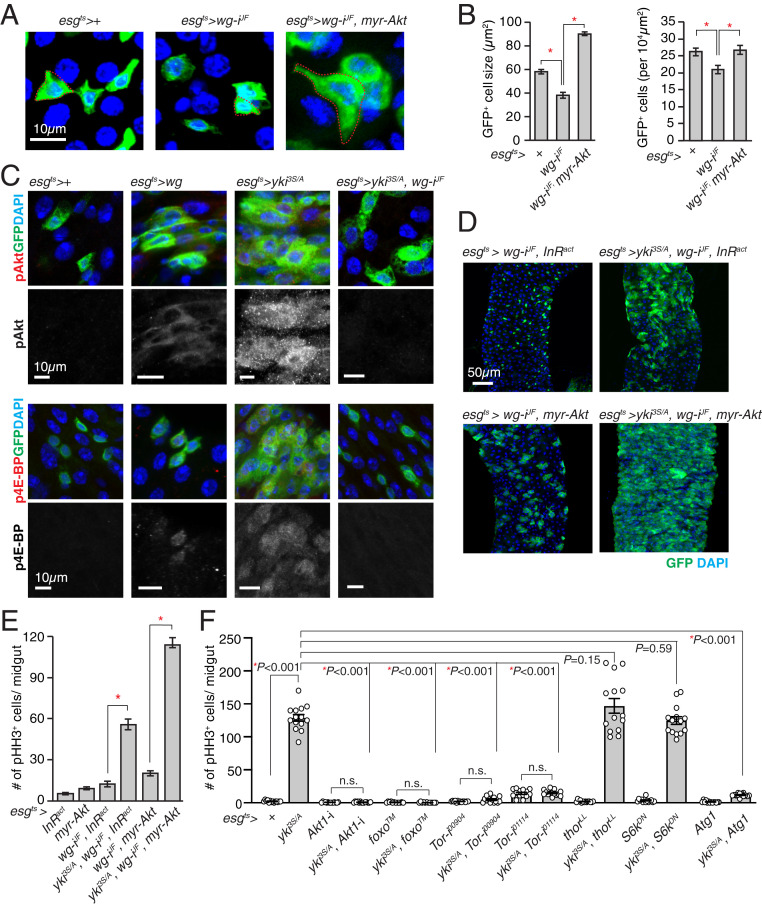
Activation of insulin/IGF signaling rescues the *yki*^*3S/A*^ tumor growth defect caused by *wg* depletion. (*A*) Representative images of *esg*^*+*^ cells. The red dotted line indicates the cell boundary. (*B*) Quantification of cell size and number. The size and number of *esg*^*+*^ cells in 100 × 100 µm^2^ were quantified. (*C*) Phospho-Akt (pAkt) and phospho-4E-BP (p4E-BP) immunostaining. Transgenes were expressed for 5 d. (Scale bar, 10 µm.) (*D*) Representative images of posterior midguts after 5 d of transgene expression with *esg*^*ts*^. GFP (green) marks *esg*^*+*^ cells, and pHH3 signals are shown in red. (Scale bar, 50 µm.) (*E*) Quantification of pHH3^+^ cells in midguts. *n* = 9 (*esg*^*ts*^ *> InR*^*act*^), *n* = 20 (*esg*^*ts*^ *> myr-Akt1*), *n* = 13 (*esg*^*ts*^ *> wg-i*^*JF*^*, InR*^*act*^), *n* = 19 (*esg*^*ts*^ *> yki*^*3S/A*^, *wg-i*^*JF*^*, InR*^*act*^), *n* = 17 (*esg*^*ts*^ *> wg-i*^*JF*^*, myr-Akt*1), *n* = 11 (*esg*^*ts*^ *> yki*^*3S/A*^, *wg-i*^*JF*^*, myr-Akt1*) biological replicates. (*F*) Quantification of pHH3^+^ cells per midgut after 5 d of transgene expression. For *B*, *E*, and *F*, mean ± SEMs are shown. *P < 0.01, two-tailed unpaired Student’s *t* test between two genotypes indicated by bracket. See also *SI Appendix*, Fig. S4.

Given the complete rescue of the tumor-growth defect by *ImpL2* depletion, we hypothesized that Wg supports *yki*^*3S/A*^ tumor growth by mainly negating the action of ImpL2. It has been shown that Wg expressed in disk tumors increases insulin/IGF signaling by increasing the expression of *insulin-like peptide receptor* (*InR*) (36). Thus, we explored whether Wg could affect insulin/IGF signaling in intestinal ISCs and EBs. While we were testing the effect of *wg* knockdown, we noticed that expression of *wg* RNAi with *esg*^*ts*^ significantly reduced the size and the number of *esg*^*+*^ cells (Fig. 2 *A* and *B*). If these phenotypes were mediated by a reduction in insulin/IGF signaling, augmenting insulin/IGF signaling in ISCs and EBs should reverse the phenotypes. Indeed, expression of a constitutively active *Akt1* (*myr-Akt1*) with *esg*^*ts*^ rescued the phenotypes caused by *wg* depletion in ISCs and EBs (Fig. 2 *A* and *B*). Next, we tested whether increasing Wg was sufficient to induce an elevation in insulin/IGF signaling in ISCs and EBs. Ectopic expression of Wg with *esg*^*ts*^ caused an increase in phospho-Akt1 and phospho-4E-BP (Thor in *Drosophila*) signals in *esg*^*+*^ cells (Fig. 2*C*). Altogether, these results suggest that Wg produced from *esg*^*+*^ cells plays an important role in regulating insulin/IGF signaling in ISCs and EBs, and ectopic Wg expression is sufficient to increase Akt1 phosphorylation in ISCs and EBs.

Previously, it was shown that phospho-Akt1 levels were increased in *yki*^*3S/A*^ tumors relative to control midguts, while phospho-Akt1 levels in the host muscle, ovaries, and heads were significantly decreased in flies bearing *yki*^*3S/A*^ tumors in the midgut (5). In accordance with the previous observations, phospho-Akt1 signals were increased in *yki*^*3S/A*^ cells compared to control cells (Fig. 2*C*). Notably, we found that *wg* depletion in *yki*^*3S/A*^ cells significantly reduced phospho-Akt1 signals, suggesting that the increase in phospho-Akt1 signals in *yki*^*3S/A*^ cells was dependent on Wg (Fig. 2*C*). If the role of Wg in supporting *yki*^*3S/A*^ tumor growth is to oppose the effect caused by ImpL2 elevation via increasing insulin/IGF signaling, augmenting insulin/IGF signaling in *yki*^*3S/A*^ cells should be sufficient to rescue the growth defect caused by *wg* depletion. Of significance, ectopic expression of either an active form of *InR* (*InR*^*act*^) or *myr-Akt1* rescued the defect in *yki*^*3S/A*^ tumor growth caused by *wg* depletion (Fig. 2 *D* and *E*). These results demonstrate that Wg is necessary for increasing insulin/IGF signaling in *yki*^*3S/A*^ tumors, which is important for negating the action of ImpL2.

### Activation of Foxo or Atg1 Attenuates *yki*^3S/A^ Tumor Growth.

Since our observations indicate that Wg supports *yki*^*3S/A*^ tumor growth by increasing insulin/IGF signaling, we decided to investigate which branch of the insulin/IGF pathway is essential for *yki*^*3S/A*^ tumor growth. Similarly, *Akt1* depletion in *yki*^*3S/A*^ cells led to a complete suppression of *yki*^*3S/A*^ tumor growth (Fig. 2*F* and *SI Appendix*, Fig. S4). Interestingly, expression of *Akt1* RNAi in combination of *yki*^*3S/A*^ with *esg*^*ts*^ almost completely eliminated *esg*^*+*^ cells, while expression of *Akt1* RNAi alone with *esg*^*ts*^ didn’t significantly alter the number of *esg*^*+*^ cells (*SI Appendix*, Fig. S4). Ectopic expression of a mutant foxo (foxo^TM^) which cannot be phosphorylated by Akt1 (37) or depletion of *Tor* significantly decreased the division of *yki*^*3S/A*^ cells (Fig. 2*F* and *SI Appendix*, Fig. S4*A*). To elucidate the Tor downstream mediator that is essential for *yki*^*3S/A*^ tumor growth, we manipulated three well-characterized Tor downstream players. Expression of either a mutant *thor* (*thor*^*LL*^) which cannot be inhibited by Tor due to the mutations at the mTOR phosphorylation sites (38) or a dominant-negative S6k (S6k^DN^) (39) in *yki*^*3S/A*^ cells did not significantly alter the division of *yki*^*3S/A*^ cells (Fig. 2*F* and *SI Appendix*, Fig. S4*A*). Of significance, ectopic expression of Atg1 in *yki*^*3S/A*^ cells almost completely abolished *yki*^*3S/A*^ tumor growth (Fig. 2*F* and *SI Appendix*, Fig. S4*A*). These results suggest that attenuation of the Foxo and Atg1 signaling branches in the insulin/IGF pathway is critical for supporting *yki*^*3S/A*^ tumor growth.

### *Wg* Is Specifically Up-Regulated in Tumors with Elevated *ImpL2* Expression.

Our observations suggest that *yki*^*3S/A*^ tumor growth is dependent on Wg up-regulation, which might restrain Foxo signaling and Atg1 signaling by antagonizing the action of ImpL2. Interestingly, a recent study demonstrated that Atg1 could inhibit Yki by direct phosphorylation (40), raising the possibility that elevation of Wg could be a mechanism specifically applicable to *yki*^*3S/A*^ tumors. Considering the fundamental role of Yki in controlling the growth of tumors (41, 42), Wg up-regulation might be a general mechanism to support the growth of tumors, especially with elevated ImpL2 expression. Thus, we decided to test whether a similar mechanism exists to support the growth of other types of midgut tumors.

**Fig. 3.**
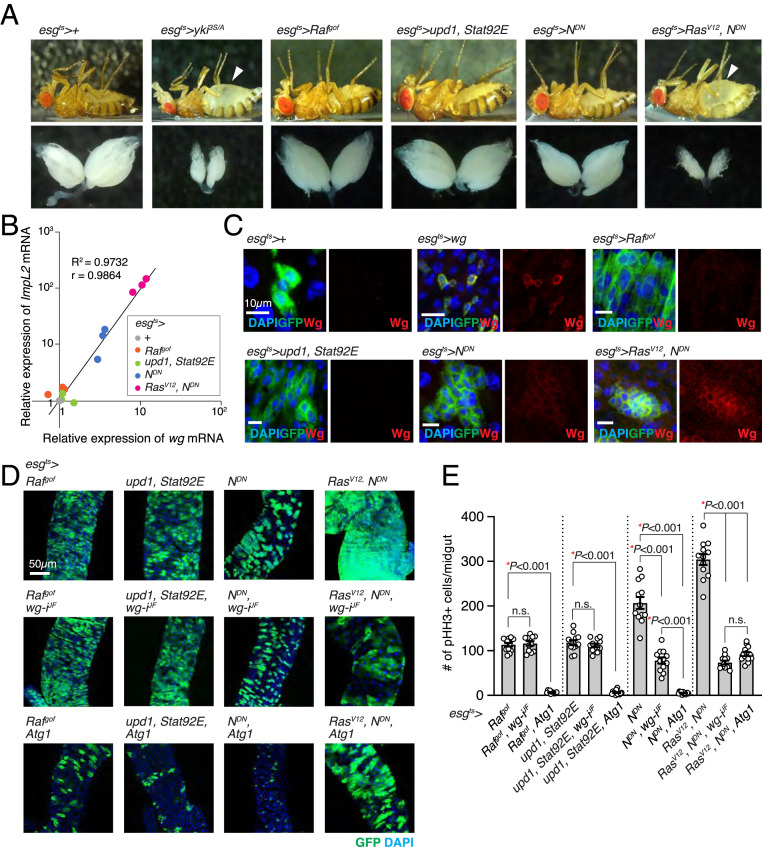
Wg is specifically required for the growth of midgut tumors with elevated ImpL2 expression. (*A*) Representative images of fly and ovary. The arrowheads indicate abdominal bloating. *esg*^*ts*^>*Ras*^*V12*^*, N*^*DN*^ flies were incubated at 29 °C for 4 d, and control (*esg*^*ts*^>+) and other flies were incubated for 6 d to induce transgene expression. (*B*) Correlative plot of relative *ImpL2* and *wg* mRNA levels. mRNA expression values of *wg* and *ImpL2* in the midguts with indicated genotypes were measured by qRT-PCR and then normalized to those values in the control midguts (*esg*^*ts*^*>*+). The relative *wg* and *ImpL2* mRNA levels from three independent experiments are shown in the *x*-axis and *y*-axis, respectively. The coefficient of determination (R^2^) is 0.9732, and the Pearson correlation coefficient (r) is 0.9864 (*P* < 0.0001). (*C*) Wg immunostaining in the midguts. (Scale bars, 10 µm.) (*D*) Images of posterior midguts. (Scale bar, 50 µm.) (*E*) Quantification of pHH3^+^ cells per midgut. Mean ± SEMs are shown with individual data points. *P < 0.01, two-tailed unpaired Student’s *t* test between two groups indicated with bracket. All transgenes were induced with *esg*^*ts*^ for 6 d except for “*Ras*^*V12*^*, N*^*DN*^*,*” “*Ras*^*V12*^*, N*^*DN*^*, wg-i*^*JF*^,” and “*Ras*^*V12*^*, N*^*DN*^*, Atg1*,” which were induced for 4 d. See also *SI Appendix*, Figs. S5 and S6.

In addition to *yki*^*3S/A*^ midgut tumors, we found that midgut tumors driven by expression of a combination of *Ras*^*V12*^ and dominant-negative *Notch* (*N*^*DN*^) with *esg*^*ts*^ (*esg*^*ts*^/*UAS-N*^*DN*^; *UAS-Ras*^*V12*^/+) induced systemic organ wasting, which was manifested by the bloating syndrome phenotype, fat body degeneration, and ovary atrophy (Fig. 3*A*). In contrast, tumors driven by expression of *N*^*DN*^ alone, a gain-of-function allele of *Raf* (*Raf*^*gof*^), or *unpaired* (*Upd1*) and *signal-transducer and activator of transcription protein at 92E* (*Stat92E*) did not induce discernable wasting phenotypes (Fig. 3*A*). Consistent with the proposed role of ImpL2 in systemic organ wasting, *ImpL2* mRNA expression was increased greater than 80-fold in *Ras*^*V12*^, *N*^*DN*^ tumors compared to control midguts, while it remained unaltered in *Raf*^*gof*^ and *Upd1*, *Stat92E* tumors (Fig. 3*B*). Note that a moderate but significant increase in *ImpL2* mRNA levels was also observed with *N*^*DN*^ tumors (Fig. 3*B*). If up-regulation of Wg is a general mechanism by which tumors evade the adverse effects caused by ImpL2 elevation, Wg expression would be expected to be increased in tumors with an elevated ImpL2 expression. Accordingly, we detected a strong correlation between *wg* and *ImpL2* mRNA levels (*R*^2^ = 0.9732 and *r* = 0.9864); *wg* mRNA expression was increased specifically in *Ras*^*V12*^, *N*^*DN*^ and *N*^*DN*^ tumors, while it was unaltered in the other tumors (Fig. 3*B*). Furthermore, Wg protein signals were cell-autonomously increased in *Ras*^*V12*^, *N*^*DN*^ and *N*^*DN*^ cells (Fig. 3*C*). Of note, overexpression of Wg or ImpL2 was not sufficient to drive expression of the other gene in *Raf*^*gof*^ and *Upd1*, *Stat92E* tumors, and ImpL2 depletion could not eliminate Wg expression in *yki*^*3S/A*^ tumors (*SI Appendix*, Fig. S5 *A*–*C*). Consistent with these observations, overexpression of Wg in *Raf*^*gof*^ and *Upd1*, *Stat92E* tumors failed to induce ovary wasting and the bloating syndrome phenotype (*SI Appendix*, Fig. S5*D*). Note that overexpression of ImpL2 in *Raf*^*gof*^ and *Upd1*, *Stat92E* tumors increased ImpL2 levels significantly but less than 10-fold, which was not enough to induce the wasting phenotypes (*SI Appendix*, Fig. S5 *B* and *D*). These results suggest that the observed correlation is not likely to be achieved because Wg can directly induce ImpL2 expression or vice versa. Given the tightness of correlation, we speculate that expression of *wg* and *ImpL2* might be under the control of a common pathway.

### Wg Is Specifically Required for the Growth of Tumors with *ImpL2* Elevation.

To assess the importance of Wg on the growth of these tumors, we depleted *wg* by expressing *wg* RNAi with *esg*^*ts*^. Notably, the growth of *Raf*^*gof*^ and *Upd1*, *Stat92E* tumors was unaltered by *wg* depletion (Fig. 3 *D* and *E*). In contrast, expression of *wg* RNAi with *esg*^*ts*^ significantly suppressed the growth of both *Ras*^*V12*^, *N*^*DN*^ and *N*^*DN*^ tumors (Fig. 3 *D* and *E*); a more prominent suppression was observed with *Ras*^*V12*^, *N*^*DN*^ tumors, which expressed significantly higher levels of *ImpL2* mRNA (Fig. 3*C*). Importantly, we found that phospho-Akt1 levels were also increased in *esg*^*ts*^>*N*^*DN*^ and *esg*^*ts*^>*Ras*^*V12*^, *N*^*DN*^ midguts and reduced by *wg* depletion (*SI Appendix*, Fig. S6), suggesting that elevation in Wg levels was also important for increasing insulin signaling in these tumors. Since we could generate a few different midgut tumors in the absence of direct manipulation of Yki, we sought to address the effect of Atg1 activation on the growth of these tumors. Interestingly, ectopic expression of Atg1 with *esg*^*ts*^ significantly suppressed the growth of all the tumors (Fig. 3 *D* and *E*). Taken together, these results suggest that Wg elevation is specifically required for the growth of the midgut tumors with elevated ImpL2 expression, while attenuation of Atg1 appears to be a general requirement for the growth of midgut tumors.

### Ectopic Wg Expression in the Muscle Increases Insulin/IGF Signaling and Rescues Muscle Degeneration in the Flies Harboring *yki*^*3S/A*^ Midgut Tumors.

*yki*^*3S/A*^ tumors in the midgut induce muscle degeneration, which is dependent on tumor-derived ImpL2 (5). Given the observation that Wg expression with *esg*^*ts*^ could increase Akt1 phosphorylation in *esg*^*+*^ cells (Fig. 2*C*), we sought to address whether ectopic expression of Wg in the muscle could rescue muscle degeneration induced by *yki*^*3S/A*^ tumors in the midgut. To express Wg in the muscle while simultaneously inducing *yki*^*3S/A*^ tumors in the midgut, we established a LexA::GAD-based temperature-sensitive inducible system [*StanEx*^*SX-4*^ (43), *LexAop-mCD8::GFP*, *tub-GAL80*^*ts*^, hereafter referred as *esg-LexA::GAD*^*ts*^, see *Materials and Methods*] and a transgenic line harboring *LexAOP-yki*^*3S/A*^. As a result, we were able to generate *yki*^*3S/A*^ tumors (*esg-LexA::GAD*^*ts*^*/+; LexAOP-yki*^*3S/A*^*/+*) independent of a GAL4/UAS system (*SI Appendix*, Fig. S4*A*). To manipulate gene expression in the muscle, we used *Mhc.F3-580-GAL4*, which has been shown to express GAL4 mainly in the adult indirect flight muscle (44).

**Fig. 4.**
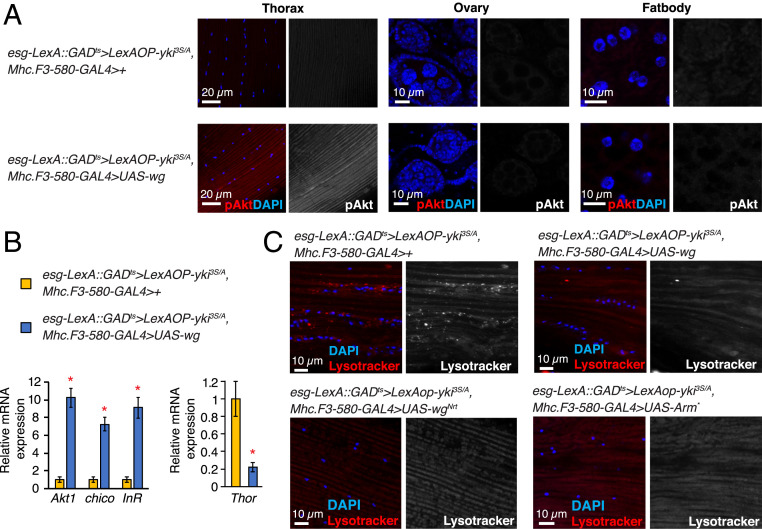
Ectopic Wg expression in the muscle increases insulin/IGF signaling. (*A*) Phospho-Akt staining. The thoraces from male flies and the ovaries and fat body from female flies were used. Phospho-Akt signals are shown in red in merge, and nuclei are stained with DAPI (blue). (*B*) Relative mRNA expression in the thorax. Mean ± SEMs are shown. *P < 0.01, two-tailed unpaired Student’s *t* test. (*C*) Lysotracker staining in thorax. Lysotracker signals are shown in red in merged images, and nuclei are stained with DAPI (blue). (Scale bar, 10 µm.) All transgenes were induced for 8 d. See also *SI Appendix*, Figs. S7 and S8.

It has been previously shown that *yki*^*3S/A*^ midgut tumors caused a disparity in insulin/IGF signaling between *yki*^*3S/A*^ tumors and host tissues in an ImpL2-dependent manner: phospho-Akt signals were significantly reduced in host tissues, while they were increased in *yki*^*3S/A*^ tumors (5). Wg expression using *Mhc.F3-580-GAL4* while inducing *yki*^*3S/A*^ tumors in the midgut (*esg-LexA::GAD*^*ts*^*/Mhc.F3-580-GAL4; LexAOP-yki*^*3S/A*^*/UAS-wg*) increased phospho-Akt signals specifically in the muscle; phospho-Akt signals in ovaries, fat body, and the neighboring muscle compartment were unaltered (Fig. 4*A* and *SI Appendix*, Fig. S7 *A* and *B*). Notably, we found that Wg expression with *Mhc.F3-580-GAL4* significantly increased mRNA levels of not only *InR* but also *Akt1* and *chico* in the muscle (Fig. 4*B*), which might contribute to the increase in Dilp sensitivity in the muscle (36, 45). Expression of Wg in the muscle had no effect on the growth of *yki*^*3S/A*^ tumors in the midgut (*SI Appendix*, Fig. S7*C*). Thus, our observations suggest that ectopic expression of Wg is sufficient to increase insulin/IGF signaling in the muscle.

A reduction in insulin/IGF signaling can lead to an activation of both Foxo and Atg1 in host tissues. Although Foxo signaling has been shown to be up-regulated in the muscle of the flies bearing *yki*^*3S/A*^ tumors (5), it is not known whether Atg1 signaling is also activated in the host tissues. Strong lysotracker signals were detected in the host tissues prepared from *yki*^*3S/A*^ tumor-bearing flies (*SI Appendix*, Fig. S8). Depleting *ImpL2* in *yki*^*3S/A*^ tumors was sufficient to suppress the accumulation of lysotracker signals in the host tissues (*SI Appendix*, Fig. S8), suggesting that induction of autophagy in the host tissues was dependent on ImpL2 derived from *yki*^*3S/A*^ tumors. Of significance, Wg expression in the muscle reduced lysotracker signals in the muscle of *yki*^*3S/A*^ tumor-bearing flies, an indicative of attenuation of Atg1 signaling (Fig. 4*C*). Additionally, Wg expression in the muscle of *yki*^*3S/A*^ tumor-bearing flies significantly reduced *thor* mRNA levels (Fig. 4*B*). Our results indicate that ectopic expression of Wg in muscle could decrease both Foxo and Atg1 activities.

**Fig. 5.**
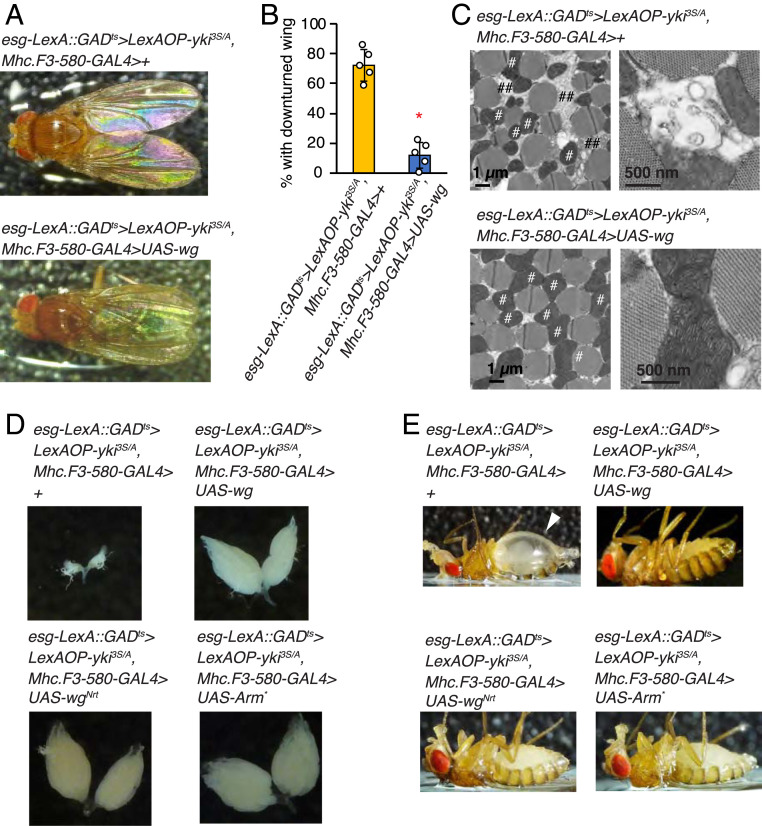
Ectopic Wg expression rescues muscle degeneration caused by *yki*^*3S/A*^ tumors in the midgut. (*A*) Dorsal view of male flies. (*B*) Penetrance of downturned wing phenotype. *n* = 69 (*esg-LexA::GAD*^*ts*^*/Mhc.F3-580-GAL4; LexAOP-yki*^*3S/A*^*/+*), *n* = 71 (*LexA::GAD*^*ts*^*/Mhc.F3-580-GAL4; LexAOP-yki*^*3S/A*^*/UAS-wg*); male flies were used for five independent experiments. Mean ± SEMs are shown with individual data points. *P < 0.01, two-tailed unpaired Student’s *t* test. (*C*) Electron microscopic images of the transverse section of indirect flight muscles. # denotes mitochondria; ## denotes low electron-dense sector between myofibers. (*D*) Representative images of ovary. (*E*) Representative images of flies. The arrowhead indicates abdominal bloating. All transgenes were induced for 8 d. See also *SI Appendix*, Fig. S7.

Strikingly, increasing Wg in the muscle was sufficient to inhibit muscle degeneration in *yki*^*3S/A*^ tumor-bearing flies, manifested by downturned wing phenotype and muscle mitochondrial fragmentation (Fig. 5 *A*–*C*). Wg expression in the muscle also rescued ovary atrophy and the bloating syndrome phenotype induced by *yki*^*3S/A*^ tumors (Fig. 5 *D* and *E*). Since Wg is a secreted protein, Wg produced in the muscle could diffuse out to act on other host tissues. To address whether activation of Wg signaling in the muscle is responsible for the rescue of the wasting phenotypes observed outside the muscle, we expressed the membrane-tethered form of Wg (Wg^Nrt^) and a truncated form of Armadillo (Arm*), which constitutively activates the Wg pathway, in the muscle to activate Wg signaling in a tissue-autonomous manner (46, 47). Of significance, expression of either Wg^Nrt^ or Arm* in the muscle was sufficient to rescue ovary atrophy and the bloating syndrome phenotype caused by having *yki*^*3S/A*^ tumors in the midguts (Fig. 5 *D* and *E*). Note that expression of either Wg^Nrt^ or Arm* in the muscle did not suppress *yki*^*3S/A*^ tumors in the midguts (*SI Appendix*, Fig. S7*C*). Thus, these results demonstrate that augmenting Wg signaling in the muscle can rescue not only muscle degeneration but also a few wasting phenotypes observed outside of muscle.

## Discussion

In this study, we demonstrate that Wg up-regulation is the mechanism by which *yki*^*3S/A*^ tumors evade the growth-impeding effects induced by ImpL2. Our observations indicate that the main role of Wg in supporting the growth of *yki*^*3S/A*^ tumors is to increase insulin/IGF signaling in a tumor-autonomous manner. Therefore, without Wg, *yki*^*3S/A*^ tumors are influenced by the action of ImpL2, which can be attenuated by either depleting *ImpL2* or augmenting insulin/IGF signaling in *yki*^*3S/A*^ tumors (Figs. 1 *C* and *D* and 2 *D* and *E*). Of significance, we observed a strong correlation between Wg and ImpL2 expression levels in several types of midgut tumors; Wg expression was increased in only those tumors with elevated *ImpL2* expression (Fig. 3 *B* and *C*). Notably, *wg* depletion specifically affected the growth of the midgut tumors with elevated ImpL2 expression (Fig. 3 *D* and *E*), suggesting that up-regulation of Wg might be a general mechanism for supporting the growth of tumors with elevated ImpL2 expression.

The formation of *yki*^*3S/A*^ tumors in the midgut induces a disparity in insulin/IGF signaling between *yki*^*3S/A*^ tumors and host tissues (5). This disparity is proposed to be the mechanistic basis for the bias in glucose metabolism between tumors and host tissues, which can be in support of tumor growth by allowing more glucose to be available to tumors (5). ImpL2 plays an essential role in inducing the disparity in insulin/IGF signaling by reducing systemic insulin/IGF signaling. Notably, depleting *ImpL2* in *yki*^*3S/A*^ tumors diminishes the bias in glucose utilization between tumors and host tissues (5). Based on our findings described in this study, we propose that Wg up-regulation in *yki*^*3S/A*^ tumors is also a crucial factor for inducing the disparity in insulin/IGF signaling. This disparity increases Foxo and Atg1 activities in the host tissues even though the role of Foxo and Atg1 in cachexia-like wasting still needs to be addressed in *Drosophila*. Nevertheless, our observations suggest that Wg-mediated up-regulation of insulin/IGF signaling might be important for restraining both Foxo and Atg1 activities in *yki*^*3S/A*^ tumors, which is critical for supporting tumor growth (Fig. 2*F* and *SI Appendix*, Fig. S2*A*).

We demonstrate that ectopic expression of Wg in the muscle rescues muscle degeneration in *yki*^*3S/A*^ tumor-bearing flies. Wg expression in the muscle increased Akt1 phosphorylation specifically in the muscle compartment but not in ovaries or fat body (Fig. 4*A*). Additionally, expression of Wg with *esg*^*ts*^ increased Akt1 phosphorylation in *esg*^*+*^ cells, not in the neighboring ECs (Fig. 2*C*). This cell-autonomous action of Wg might account for the rescue of muscle degeneration by Wg expression (Fig. 5 *A*–*C*). It is intriguing to note that augmenting Wg in the muscle also rescued ovary degeneration and the bloating syndrome phenotype (Fig. 5 *D* and *E*). The results obtained with expression of Wg^Nrt^ and Arm* in the muscle defy the possibility that Wg diffused out from the muscle is responsible for the rescue of the wasting phenotypes observed outside the muscle (Fig. 5 *D* and *E*). Expression of Wg^Nrt^ and Arm* in muscle should have turned on Wg signaling in a tissue-autonomous manner (46, 47). Thus, our observations imply the importance of the muscle in inducing the systemic wasting phenotypes observed in flies bearing *yki*^*3S/A*^ midgut tumors. We speculate that potential interorgan communication between degenerating muscle and other tissues might be the basis of the Wg-mediated rescue of the wasting phenotypes outside the muscle.

Taken together, our study provides insights into the mechanism by which tumors overcome the action of the tumor-derived wasting factor ImpL2. Interestingly, the mammalian ImpL2-like proteins IGFBPs are up-regulated in multiple types of cancers, and several IGFBPs are shown to function as tumor suppressors in mammals (48–52). Considering that the conserved role of Wnt/Wg signaling in increasing IGF signaling (53–55), it would be intriguing to investigate whether a similar relationship between Wnt and IGFBPs exists in human cancers. If such a relationship is present in humans, targeting Wnt/Wg signaling could be more effective for treating a subtype of cancers with elevated IGFBP levels. Additionally, our findings suggest a principle for designing an efficient strategy to counter muscle wasting in cancer patients without augmenting tumor growth. In mammals, muscle wasting during cancer cachexia is associated with a decrease in IGF signaling (11–13). Interestingly, a recent study showed that expressing Wnt7a in muscle could suppress muscle wasting in a mouse model of cancer cachexia (56). Our findings further support that increasing Wnt/Wg signaling in the muscle could be an efficient strategy to treat muscle wasting. Given the important role of Wnt/Wg signaling in tumor growth and progression, it can be foreseen that increasing Wnt7a could lead to the deleterious effects caused by promotion of tumor growth and progression. Therefore, it would be interesting to test if a membrane-tethered form of Wnt7a could be designed and delivered specifically to the muscle to make the strategy safer.

## Materials and Methods

### Fly Husbandry and Genetics.

Several lines of the fruit fly *Drosophila melanogaster* were used in this study and are listed in *SI Appendix*, Table S1. Fly crosses were set up in vials containing standard molasses agar medium, kept at room temperature for 3 d, and then transferred to 18 °C to restrict the expression of GAL4-induced transgenes throughout the development. Adult progenies were collected and incubated at 29 °C for 3 to 8 d prior to dissection to induce the transgenes. During incubation at 29 **°**C, flies were transferred onto fresh food every 2 d. Female flies (<16 d old) were used for all experiments except for those employed in thorax, in which male flies were used instead.

To manipulate ISCs and EBs, we used *esg-GAL4, tub-GAL80*^*ts*^, *UAS-GFP* (referred as *esg*^*ts*^) and *StenEx*^*SX-4*^*, tub-GAL80*^*ts*^, *LexAop-mCD8::GFP,* (referred as *esg-lexA*::*HG*^*ts*^*). StenEx*^*SX-4*^ (Bloomington Drosophila Stock Center [BDSC] no. 66659) (43) was recombined with *tub-GAL80*^*ts*^ (BDSC no. 7108) and *LexAop2-mCD8::GFP* (BDSC no. 32205) to generate *esg-lexA*::*HG*^*ts*^. Other *Drosophila* lines and their sources are listed in *SI Appendix*, Table S1.

### Generation of *LexAOP-yki*^*3S/A*^ Line.

We obtained the *yki* ^*S111A.S168A.S250A*^ (referred as *yki*^*3S/A*^) coding sequence from Dr. Kenneth Irvine at Rutgers University, Piscataway, NJ (10). The *yki*^*3S/A*^ sequence was amplified by PCR using the following primers: forward: 5′-ctc​gag​ATG​TTA​ACG​ACG​ATG​TCA​GCC​AG-3′ and reverse: 5′-tct​aga​tta​ATT​AAT​TTT​ATA​CCA​TTC​CAA​ATC​GTC​AGG-3′. The PCR product was subcloned into the pJFRC19-13XLexAOP-IVS-myr::GFP vector (Addgene no. 26224) to generate pJFRC19-13XLexAOP-*yki*^*3S/A*^. The resulting construct was targeted into the attP2 site through germline transformation.

### qRT-PCR.

The total RNA from adult female midguts and male thoraces was isolated with TRIzol (Invitrogen, category no. 15596026). RNA (1 µg) was used to produce complementary DNA (cDNA) with iScript Reverse Transcription Supermix (Bio-Rad, category no. 1725120). The cDNA was subjected to quantitative real-time PCR with iTaq Universal SYBR Green Supermix (Bio-Rad, category no. 1708840) and CFX-96 (Bio-Rad). *RpL32* was used for normalization. The fold change in RNA expression compared to the control was calculated and plotted for relative mRNA expression. Primers used for qRT-PCR are described in *SI Appendix*, Table S1.

### Antibody Staining and Immunofluorescence Microscopy.

To remove food from the midgut, flies were fed on 4% sucrose for ∼4 h prior to dissection. We prepared midguts, ovaries, and fat body from female flies and thoraxes from male flies. Tissues dissected in phosphate-buffered saline (PBS) were fixed in 4% paraformaldehyde (PFA; Electron Microscopy Sciences) for 20 min and then washed three times for 5 min each with PBST (PBS supplemented with 0.2% Triton X-100). For permeabilization and blocking, tissue samples were incubated in blocking buffer (PBST supplemented with 5% normal goat serum) for 1 h at room temperature. Then, tissue samples were incubated with primary antibody in blocking buffer overnight at 4 **°**C. The tissue samples were washed three times with PBST for 5 min each and then incubated with secondary antibody for 2 to 3 h at room temperature. Stained tissues were washed three times with PBST and mounted with Vectashield (Vector Laboratories, category no. H-1000). Fluorescence micrographs were acquired with a Leica SP8 laser scanning confocal microscope with a 40×/1.25 oil objective lens. Fiji software was used for further adjustment and assembly of the acquired images.

### Lysotracker Staining.

For lysotracker staining, the thoraces of male flies were dissected in four pieces: cut once in the sagittal section and once in the transverse section in PBS. Female flies were used for ovary staining, and the female abdominal cuticle was used for fat body staining. Freshly dissected tissues were incubated for 5 min in 50 nM LysoTracker Red DND-99 (Life Technologies, category no. L7528) in PBS, rinsed quickly three times in PBS, and then fixed in 4% PFA in PBS for 5 min in room temperature. The samples were briefly incubated in PBST for permeabilization, stained with DAPI, rinsed three times with PBST, and mounted in Vectashield. Muscle fibers were carefully dissociated and removed from the thoracic cuticle to spread flat when mounting.

### Quantification of Phospho-Histone H3–Positive Cells.

To determine the number of cells undergoing mitotic division, midguts were dissected and stained with anti-pHH3 (phospho-histone H3) antibody (Abcam, category no. ab14955). The number of pHH3-positive nuclei was counted from the entire midgut.

### Measurement of Cell Size.

The outlines of individual cells from confocal images acquired with a 40×/1.25 oil objective lens was traced, and the area was measured with Fiji software.

### Electron Microscopy.

The thoraces from male flies were dissected and fixed overnight in in 4% glutaraldehyde in 0.1 M sodium cacodylate buffer, pH 7.2. The samples were washed in buffer, postfixed in 1% osmium tetroxide for 90 min, rinsed, stained in 1% uranyl acetate, dehydrated in ethanol solutions, and embedded in epoxy resin (Epon Araldite). Serial sections (80 nm) were aligned and viewed on a JEOL-1230 transmission electron microscope with an AMT XR80 camera.

### Quantification and Statistical Analysis.

All the midgut images presented and used for quantification were obtained from the posterior R5 region of female flies, except for the pHH3-positive nuclei quantification, which was done from the entire midguts. Statistical analyses were performed using Microsoft Excel and GraphPad Prism 8. All *P* values were determined by two-tailed Student’s *t* test with unequal variances. Statistical significance was depicted by asterisks in the figures: *P < 0.01. Sample sizes were chosen empirically based on the observed effects and indicated in the figure legends.

## Supplementary Material

Supplementary File

## Data Availability

All study data are included in the article and/or *SI Appendix*.

## References

[1] F. Penna, F. M. Baccino, P. Costelli, Coming back: Autophagy in cachexia. Curr. Opin. Clin. Nutr. Metab. Care 17, 241–246 (2014). 24535215 10.1097/MCO.0000000000000048

[2] S. Peixoto da Silva, J. M. O. Santos, M. P. Costa E Silva, R. M. Gil da Costa, R. Medeiros, Cancer cachexia and its pathophysiology: Links with sarcopenia, anorexia and asthenia. J. Cachexia Sarcopenia Muscle 11, 619–635 (2020). 32142217 10.1002/jcsm.12528 PMC7296264

[3] J. M. Argilés, S. Busquets, B. Stemmler, F. J. López-Soriano, Cancer cachexia: Understanding the molecular basis. Nat. Rev. Cancer 14, 754–762 (2014). 25291291 10.1038/nrc3829

[4] V. E. Baracos, L. Martin, M. Korc, D. C. Guttridge, K. C. H. Fearon, Cancer-associated cachexia. Nat. Rev. Dis. Primers 4, 17105 (2018). 29345251 10.1038/nrdp.2017.105

[5] Y. Kwon ., Systemic organ wasting induced by localized expression of the secreted insulin/IGF antagonist ImpL2. Dev. Cell 33, 36–46 (2015). 25850671 10.1016/j.devcel.2015.02.012 PMC4437243

[6] A. Figueroa-Clarevega, D. Bilder, Malignant Drosophila tumors interrupt insulin signaling to induce cachexia-like wasting. Dev. Cell 33, 47–55 (2015). 25850672 10.1016/j.devcel.2015.03.001 PMC4390765

[7] R. E. Kreipke, Y. V. Kwon, H. R. Shcherbata, H. Ruohola-Baker, Drosophila melanogaster as a model of muscle degeneration disorders. Curr. Top. Dev. Biol. 121, 83–109 (2017). 28057309 10.1016/bs.ctdb.2016.07.003

[8] D. Chatterjee, W. M. Deng, Drosophila model in cancer: An introduction. Adv. Exp. Med. Biol. 1167, 1–14 (2019). 31520346 10.1007/978-3-030-23629-8_1

[9] P. Saavedra, N. Perrimon, Drosophila as a model for tumor-induced organ wasting. Adv. Exp. Med. Biol. 1167, 191–205 (2019). 31520356 10.1007/978-3-030-23629-8_11

[10] H. Oh, K. D. Irvine, In vivo analysis of Yorkie phosphorylation sites. Oncogene 28, 1916–1927 (2009). 19330023 10.1038/onc.2009.43 PMC2701235

[11] S. C. Bodine ., Akt/mTOR pathway is a crucial regulator of skeletal muscle hypertrophy and can prevent muscle atrophy in vivo. Nat. Cell Biol. 3, 1014–1019 (2001). 11715023 10.1038/ncb1101-1014

[12] S. Schiaffino, K. A. Dyar, S. Ciciliot, B. Blaauw, M. Sandri, Mechanisms regulating skeletal muscle growth and atrophy. FEBS J. 280, 4294–4314 (2013). 23517348 10.1111/febs.12253

[13] P. Costelli ., IGF-1 is downregulated in experimental cancer cachexia. Am. J. Physiol. Regul. Integr. Comp. Physiol. 291, R674–R683 (2006). 16614058 10.1152/ajpregu.00104.2006

[14] K. C. Fearon, D. J. Glass, D. C. Guttridge, Cancer cachexia: Mediators, signaling, and metabolic pathways. Cell Metab. 16, 153–166 (2012). 22795476 10.1016/j.cmet.2012.06.011

[15] F. Penna ., Autophagic degradation contributes to muscle wasting in cancer cachexia. Am. J. Pathol. 182, 1367–1378 (2013). 23395093 10.1016/j.ajpath.2012.12.023

[16] K. Fearon, J. Arends, V. Baracos, Understanding the mechanisms and treatment options in cancer cachexia. Nat. Rev. Clin. Oncol. 10, 90–99 (2013). 23207794 10.1038/nrclinonc.2012.209

[17] J. L. Chen ., Elevated expression of activins promotes muscle wasting and cachexia. FASEB J. 28, 1711–1723 (2014). 24378873 10.1096/fj.13-245894

[18] H. Q. Han, X. Zhou, W. E. Mitch, A. L. Goldberg, Myostatin/activin pathway antagonism: Molecular basis and therapeutic potential. Int. J. Biochem. Cell Biol. 45, 2333–2347 (2013). 23721881 10.1016/j.biocel.2013.05.019

[19] X. Zhou ., Reversal of cancer cachexia and muscle wasting by ActRIIB antagonism leads to prolonged survival. Cell 142, 531–543 (2010). 20723755 10.1016/j.cell.2010.07.011

[20] S. K. Das ., Adipose triglyceride lipase contributes to cancer-associated cachexia. Science 333, 233–238 (2011). 21680814 10.1126/science.1198973

[21] S. Kir ., Tumour-derived PTH-related protein triggers adipose tissue browning and cancer cachexia. Nature 513, 100–104 (2014). 25043053 10.1038/nature13528 PMC4224962

[22] Y. S. Gallot ., Myostatin gene inactivation prevents skeletal muscle wasting in cancer. Cancer Res. 74, 7344–7356 (2014). 25336187 10.1158/0008-5472.CAN-14-0057

[23] D. R. Nässel, Y. Liu, J. Luo, Insulin/IGF signaling and its regulation in Drosophila. Gen. Comp. Endocrinol. 221, 255–266 (2015). 25616197 10.1016/j.ygcen.2014.11.021

[24] Y. Y. Chang, T. P. Neufeld, Autophagy takes flight in Drosophila. FEBS Lett. 584, 1342–1349 (2010). 20079355 10.1016/j.febslet.2010.01.006 PMC2843783

[25] M. S. Dionne, L. N. Pham, M. Shirasu-Hiza, D. S. Schneider, Akt and FOXO dysregulation contribute to infection-induced wasting in Drosophila. Curr. Biol. 16, 1977–1985 (2006). 17055976 10.1016/j.cub.2006.08.052

[26] J. B. Cordero, R. K. Stefanatos, A. Scopelliti, M. Vidal, O. J. Sansom, Inducible progenitor-derived Wingless regulates adult midgut regeneration in Drosophila. EMBO J. 31, 3901–3917 (2012). 22948071 10.1038/emboj.2012.248 PMC3463851

[27] G. Lin, N. Xu, R. Xi, Paracrine Wingless signalling controls self-renewal of Drosophila intestinal stem cells. Nature 455, 1119–1123 (2008). 18806781 10.1038/nature07329

[28] Z. Chen, J. Y. Zhu, Y. Fu, A. Richman, Z. Han, Wnt4 is required for ostia development in the Drosophila heart. Dev. Biol. 413, 188–198 (2016). 26994311 10.1016/j.ydbio.2016.03.008 PMC4857614

[29] J. H. Lee, R. Bassel-Duby, E. N. Olson, Heart- and muscle-derived signaling system dependent on MED13 and Wingless controls obesity in Drosophila. Proc. Natl. Acad. Sci. U.S.A. 111, 9491–9496 (2014). 24979807 10.1073/pnas.1409427111 PMC4084481

[30] B. Honegger ., Imp-L2, a putative homolog of vertebrate IGF-binding protein 7, counteracts insulin signaling in Drosophila and is essential for starvation resistance. J. Biol. 7, 10 (2008). 18412985 10.1186/jbiol72 PMC2323038

[31] R. Bader ., The IGFBP7 homolog Imp-L2 promotes insulin signaling in distinct neurons of the Drosophila brain. J. Cell Sci. 126, 2571–2576 (2013). 23591813 10.1242/jcs.120261

[32] L. Sarraf-Zadeh ., Local requirement of the Drosophila insulin binding protein imp-L2 in coordinating developmental progression with nutritional conditions. Dev. Biol. 381, 97–106 (2013). 23773803 10.1016/j.ydbio.2013.06.008

[33] N. Okamoto ., A secreted decoy of InR antagonizes insulin/IGF signaling to restrict body growth in Drosophila. Genes Dev. 27, 87–97 (2013). 23307869 10.1101/gad.204479.112 PMC3553286

[34] M. Amoyel, K. H. Hillion, S. R. Margolis, E. A. Bach, Somatic stem cell differentiation is regulated by PI3K/Tor signaling in response to local cues. Development 143, 3914–3925 (2016). 27633989 10.1242/dev.139782 PMC5117146

[35] Y. Nie ., Oncogenic pathways and loss of the Rab11 GTPase synergize to alter metabolism in *Drosophila*. Genetics 212, 1227–1239 (2019). 31213502 10.1534/genetics.119.302137 PMC6707446

[36] S. Hirabayashi, T. J. Baranski, R. L. Cagan, Transformed Drosophila cells evade diet-mediated insulin resistance through wingless signaling. Cell 154, 664–675 (2013). 23911328 10.1016/j.cell.2013.06.030 PMC3800019

[37] D. S. Hwangbo, B. Gershman, M. P. Tu, M. Palmer, M. Tatar, Drosophila dFOXO controls lifespan and regulates insulin signalling in brain and fat body. Nature 429, 562–566 (2004).Corrected in: *Nature* **434**, 118 (2005). 15175753 10.1038/nature02549

[38] M. Miron ., The translational inhibitor 4E-BP is an effector of PI(3)K/Akt signalling and cell growth in Drosophila. Nat. Cell Biol. 3, 596–601 (2001). 11389445 10.1038/35078571

[39] H. Barcelo, M. J. Stewart, Altering Drosophila S6 kinase activity is consistent with a role for S6 kinase in growth. Genesis 34, 83–85 (2002). 12324955 10.1002/gene.10132

[40] L. K. Tyra, N. Nandi, C. Tracy, H. Krämer, Yorkie growth-promoting activity is limited by Atg1-mediated phosphorylation. Dev. Cell 52, 605–616.e7 (2020). 32032548 10.1016/j.devcel.2020.01.011 PMC7105283

[41] K. Snigdha, K. S. Gangwani, G. V. Lapalikar, A. Singh, M. Kango-Singh, Hippo signaling in cancer: Lessons from *Drosophila* models. Front. Cell Dev. Biol. 7, 85 (2019). 31231648 10.3389/fcell.2019.00085 PMC6558396

[42] Y. Zheng, D. Pan, The Hippo signaling pathway in development and disease. Dev. Cell 50, 264–282 (2019). 31386861 10.1016/j.devcel.2019.06.003 PMC6748048

[43] L. Kockel ., A Drosophila LexA enhancer-trap resource for developmental biology and neuroendocrine research. G3 (Bethesda) 6, 3017–3026 (2016). 27527793 10.1534/g3.116.031229 PMC5068927

[44] K. M. Gajewski, R. A. Schulz, CF2 represses Actin 88F gene expression and maintains filament balance during indirect flight muscle development in Drosophila. PLoS One 5, e10713 (2010). 20520827 10.1371/journal.pone.0010713 PMC2876027

[45] W. Zhang, B. J. Thompson, V. Hietakangas, S. M. Cohen, MAPK/ERK signaling regulates insulin sensitivity to control glucose metabolism in Drosophila. PLoS Genet. 7, e1002429 (2011). 22242005 10.1371/journal.pgen.1002429 PMC3248469

[46] M. Zecca, K. Basler, G. Struhl, Direct and long-range action of a Wingless morphogen gradient. Cell 87, 833–844 (1996). 8945511 10.1016/s0092-8674(00)81991-1

[47] M. Zecca, G. Struhl, Recruitment of cells into the Drosophila wing primordium by a feed-forward circuit of vestigial autoregulation. Development 134, 3001–3010 (2007). 17634192 10.1242/dev.006411

[48] Y. X. Chan ., Higher IGFBP3 is associated with increased incidence of colorectal cancer in older men independently of IGF1. Clin. Endocrinol. (Oxf.) 88, 333–340 (2018). 29044573 10.1111/cen.13499

[49] K. Wu ., The role of IGFBP-5 in mediating the anti-proliferation effect of tetrandrine in human colon cancer cells. Int. J. Oncol. 46, 1205–1213 (2015). 25524807 10.3892/ijo.2014.2800

[50] J. Wang ., Insulin-like growth factor binding protein 5 (IGFBP5) functions as a tumor suppressor in human melanoma cells. Oncotarget 6, 20636–20649 (2015). 26010068 10.18632/oncotarget.4114 PMC4653031

[51] N. Wajapeyee, R. W. Serra, X. Zhu, M. Mahalingam, M. R. Green, Oncogenic BRAF induces senescence and apoptosis through pathways mediated by the secreted protein IGFBP7. Cell 132, 363–374 (2008). 18267069 10.1016/j.cell.2007.12.032 PMC2266096

[52] W. Zumkeller, IGFs and IGFBPs: Surrogate markers for diagnosis and surveillance of tumour growth? Mol. Pathol. 54, 285–288 (2001). 11577168 10.1136/mp.54.5.285 PMC1187083

[53] J. Palsgaard ., Cross-talk between insulin and Wnt signaling in preadipocytes: Role of Wnt co-receptor low density lipoprotein receptor-related protein-5 (LRP5). J. Biol. Chem. 287, 12016–12026 (2012). 22337886 10.1074/jbc.M111.337048 PMC3320948

[54] M. Abiola ., Activation of Wnt/beta-catenin signaling increases insulin sensitivity through a reciprocal regulation of Wnt10b and SREBP-1c in skeletal muscle cells. PLoS One 4, e8509 (2009). 20041157 10.1371/journal.pone.0008509 PMC2794543

[55] K. Inoki ., TSC2 integrates Wnt and energy signals via a coordinated phosphorylation by AMPK and GSK3 to regulate cell growth. Cell 126, 955–968 (2006). 16959574 10.1016/j.cell.2006.06.055

[56] M. Schmidt, C. Poser, J. von Maltzahn, Wnt7a counteracts cancer cachexia. Mol. Ther. Oncolytics 16, 134–146 (2020). 32055677 10.1016/j.omto.2019.12.011 PMC7005483

