# Supplementary material for: Tumors overcome the action of the wasting factor ImpL2 by locally elevating Wnt/Wingless
Source: Proc Natl Acad Sci U S A. 2021 Jun 2;118(23):e2020120118. doi: 10.1073/pnas.2020120118 (PMC8201939; doi:10.1073/pnas.2020120118)
Supplement: Supplementary File [file pnas.2020120118.sapp.pdf]

PNAS
www.pnas.org

Supplementary Information for

Tumors Overcome the Action of the Wasting Factor ImpL2 by Locally Elevating Wnt/Wingless

Jiae Lee[1], Katelyn G.-L. Ng[1], Kenneth M. Dombek[1], Dae Seok Eom[2], and Young V. Kwon[1,*]

[1]Department of Biochemistry, School of Medicine, University of Washington, Seattle, WA 98195, USA
[2]Department of Developmental and Cell Biology, School of Biological Sciences, University of California, Irvine, CA 92697, USA

Corresponding author: Young V. Kwon
Email: ykwon7@uw.edu

This PDF file includes:

Table S1
Figures S1 to S8

Table S1. List of reagents

REAGENT or RESOURCE	SOURCE	IDENTIFIER
Antibodies		
anti-Wg (Mouse monoclonal)	Developmental Studies Hybridoma Bank	Cat# 4d4; RRID: AB_528512
anti-Histone H3 (phospho S10) (Mouse monoclonal)	Abcam	Cat# ab14955; RRID: AB_443110
anti-GFP antibody, Alexa488 (Rabbit polyclonal)	Thermo Fisher Scientific	Cat# A21311; RRID: AB221477
anti-Phospho-Akt (Ser473) (Rabbit polyclonal)	Cell Signaling Technology	Cat# 9271; RRID: AB_329825
Goat anti-Mouse IgG, Alexa594	Thermo Fisher Scientific	Cat# A-11005; RRID: AB2534073
Goat anti-Rabbit IgG, Alexa594	Thermo Fisher Scientific	Cat# A-11012, RRID: AB_ 2534079
Chemicals, Peptides, and Recombinant Proteins		
16% paraformaldehyde	Electron Microscopy Sciences	Cat# RT15710
TRIzol™ Reagent	Invitrogen	Cat# 15596026
LysoTracker™ Red DND-99	Life Technologies	Cat# L7528
DAPI	Sigma-Aldrich	Cat# D9542
Vectashield	Vector Laboratories	Cat# H-1000
Critical Commercial Assays		
iTaq™ Universal SYBR Green Supermix	Bio-Rad	Cat#1725120
iScript™ Reverse Transcription Supermix for RT-PCR	Bio-Rad	Cat#1708840
Experimental Models: Organisms/Strains		
D. melanogaster: esg-GAL4, tub-GAL80^{ts}, UAS-GFP	Lab stock	N/A
D. melanogaster: esg-LexA::GAD, tub-GAL80^{ts}, lexAop-GFP; MKRS/TM6B	This paper	N/A
D. melanogaster: esg-LexA::GAD, tub-GAL80^{ts}, lexAop-GFP; UAS-wg-HA	This paper	N/A
D. melanogaster: w[*]; P{y[+t7.7] w[+mC]=UAS-yki.S111A.S168A.S250A.V5}attP2	Bloomington Drosophila Stock Center	BDSC:28817; FlyBase: FBtp0051046
D. melanogaster: Mhc-GAL4.F3-580/CyO; {LexAop-yki.S111A.S168A.S250A.V5}attP2	This paper	N/A

D. melanogaster: RNAi of wg: y[1] v[1]; P{y[+t7.7] v[+t1.8]=TRiP.JF01257}attP2	Bloomington Drosophila Stock Center	BDSC:31310; FlyBase: FBtp0054819
D. melanogaster: RNAi of wg: y[1] sc[*] v[1] sev[21]; P{y[+t7.7] v[+t1.8]=TRiP.HMS00794}attP2	Bloomington Drosophila Stock Center	BDSC:32994; FlyBase: FBtp0065055
D. melanogaster: RNAi of ImpL2: UAS-ImpL2 RNAi	National Institute of Genetics (NIG-Fly)	NIG: 15009R-3; FlyBase: FBtp0074948
D. melanogaster: UAS-myr-Akt/TM3,Sb	Bloomington Drosophila Stock Center	BDSC:50758
D. melanogaster: w[*]; P{w[+mC]=UAS-wg.H.T:HA1}6C	Bloomington Drosophila Stock Center	BDSC:5918; FlyBase: FBtp0008631
D. melanogaster: UAS-InR^{ACT}	Lab stock	N/A
D. melanogaster: RNAi of Akt1: y[1] v[1]; P{y[+t7.7] v[+t1.8]=TRiP.HMS00007}attP2	Bloomington Drosophila Stock Center	BDSC:33615; FBtp0064637
D. melanogaster: RNAi of Tor: y[1] sc[*] v[1] sev[21]; P{y[+t7.7] v[+t1.8]=TRiP.HMS00904}attP2	Bloomington Drosophila Stock Center	BDSC:33951; FBtp0065159
D. melanogaster: RNAi of Tor: y[1] sc[*] v[1] sev[21]; P{y[+t7.7] v[+t1.8]=TRiP.HMS00904}attP2	Bloomington Drosophila Stock Center	BDSC:34639; FBtp0065357
D. melanogaster: w[*]; P{w[+mC]=UAS-thor.LL}s/TM6C, cu[1] Sb[1]	Bloomington Drosophila Stock Center	BDSC:24854; FBtp0017257
D. melanogaster: w[1118]; P{w[+mC]=UAS-S6K.KQ}2	Bloomington Drosophila Stock Center	BDSC:6911; FBtp0016408
D. melanogaster: y[1] w[*]; P{w[+mC]=UAS-Atg1.S}6B	Bloomington Drosophila Stock Center	BDSC:51655; FBtp0041043
D. melanogaster: UAS-foxo-TM	Lab stock	FBtp0017932
D. melanogaster: UAS-Upd1, UAS-Stat92E	Gift from G.H.Baeg	N/A
D. melanogaster: UAS-Notch^{DN}/CyO; UAS-Ras^{V12}/TM6B	Lab stock	N/A
D. melanogaster: UAS-Notch^{DN}	Lab stock	N/A
D. melanogaster: y w F122; FRT42 pwn/CyO; UAS>>wg^{Nrt} [W147.1F] /TM2	Gift from Gary Struhl	N/A
D. melanogaster: y w 5XQE.DsRed, w+; hsp70.GFP, y+ FRT39 ap; UAS>>Arm* [AM782.6F] /SM5-TM6B	Gift from Gary Struhl	N/A
Oligonucleotides		

Primers for *wg*:<br>Forward: 5’- CCAAGTCGAGGGCAAACAGAA -3’<br>Reverse: 5’- TGGATCGCTGGGTCCATGTA -3’	FlyPrimerBank	PP10700
Primers for *ImpL2*:<br>Forward: 5’-AAGAGCCGTGGACCTGGTA-3’<br>Reverse: 5’-TTGGTGAACTTGAGCCAGTCG-3’	Kwon et al., 2015 (1)	N/A
Primers for *Akt1*:<br>Forward: 5’-CCCAGCGTTACATCGGGTC-3’<br>Reverse: 5’-GCTCGCCCCTCTTCATCAG-3’	Kwon et al., 2015 (1)	N/A
Primers for *chico*:<br>Forward: 5’-GCGCACTCACCTTATGACCA-3’<br>Reverse: 5’-GCACACGAATGTCAGGGATTT-3’	Kwon et al., 2015 (1)	N/A
Primers for *InR*:<br>Forward: 5’-CCGCAAGCAGTGAAGAAGC-3’<br>Reverse: 5’-CGTCGTCTCCACTTCGTCAAA-3’	FlyPrimerBank	PP25348
Primers for *Thor*:<br>Forward: 5’- CAGGAAGGTTGTCATCTCGGA -3’<br>Reverse: 5’- GGAGTGGTGGAGTAGAGGGTT-3’	FlyPrimerBank	PP5424
Primers for *RpL32*:<br>Forward: 5’- GCTAAGCTGTCGCACAAATG-3’<br>Reverse: 5’- GTTCGATCCGTAACCGATGT-3’	Kwon et al., 2015 (1)	N/A
Software and Algorithms		
Fiji	ImageJ	http://fiji.sc/
Prism 8.0	GraphPad	www.graphpad.com
Leica Application Suite X (LAS X) software	Leica	RRID:SCR_013673

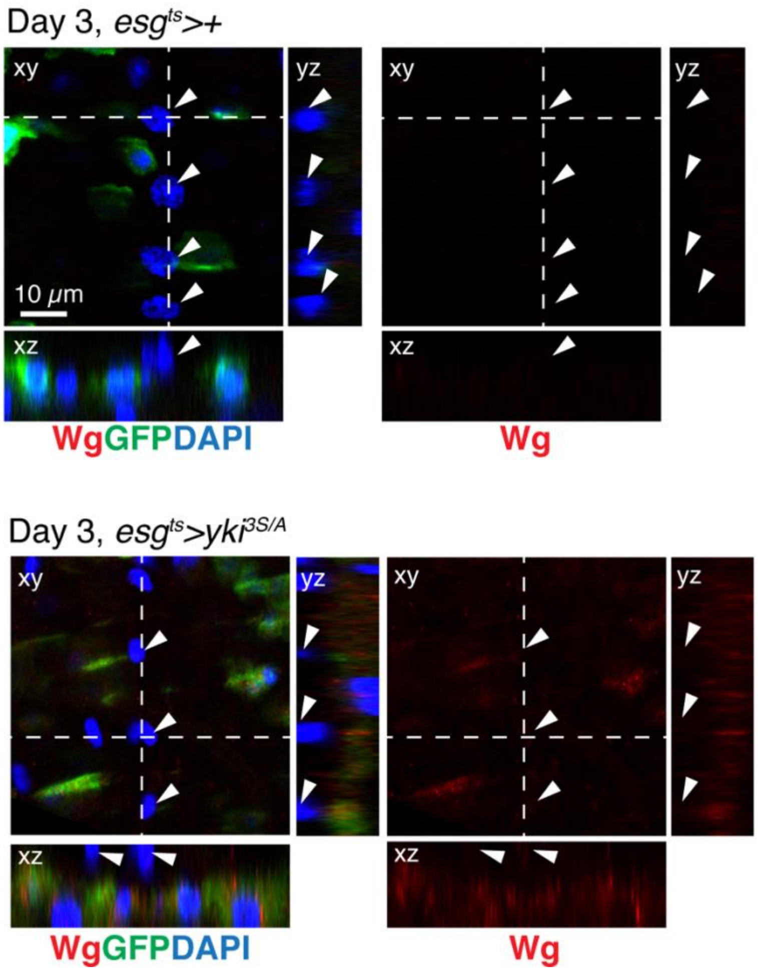

Figure S1. ***yki$^{3S/A}$*** **tumors do not increase Wg in visceral muscles.**

Immunostaining of Wg in posterior midguts, shown with orthogonal views. Transgenes were induced for 3 days with *esg^{ts}*. *esg^{+}* cells are marked by GFP (green), Wg staining is shown in red, and nuclei are stained with DAPI (blue) in merged images. Arrowheads point to nuclei of visceral muscle. Scale bar, 10 μm.

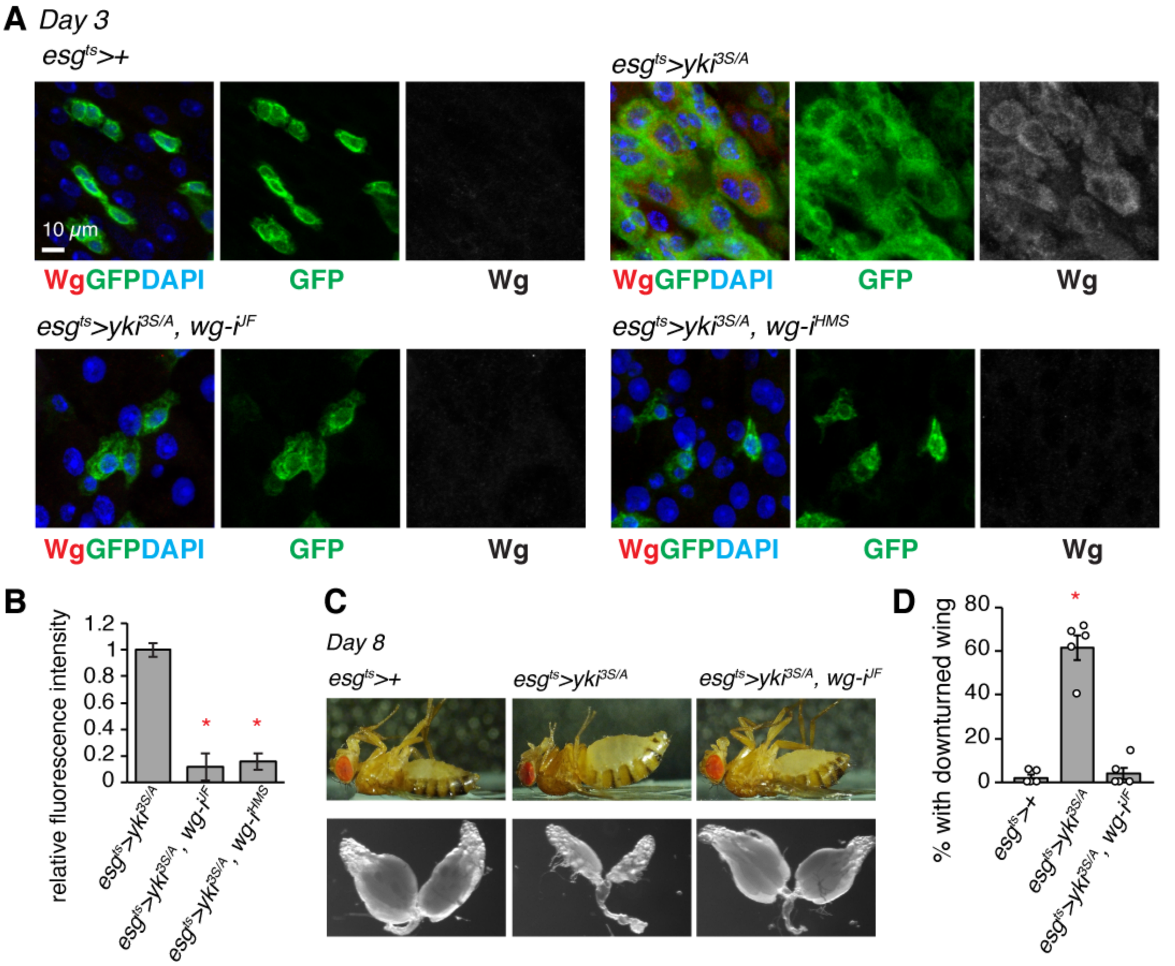

Figure S2. Wg depletion in *yki*$^{3S/A}$ cells suppresses tumor induced wasting.

(A) Wg immunostaining**.** Scale bars, 10 µm.

(B) Quantification of *wg RNAi* efficiency. *wg-i*^{JF} (JF01257) or *wg-i*^{HMS} (HMS00794) reduced Wg levels to 11.7 ± 10.7% or 16.1 ± 6%, respectively, in *yki*$^{3S/A}$ cells.

(C) Representative images of fly and ovaries.

(D) Quantification of downturned wing phenotype. Mean±SEMs are shown. **P*<0.01, two-tailed unpaired Student's *t*-test.

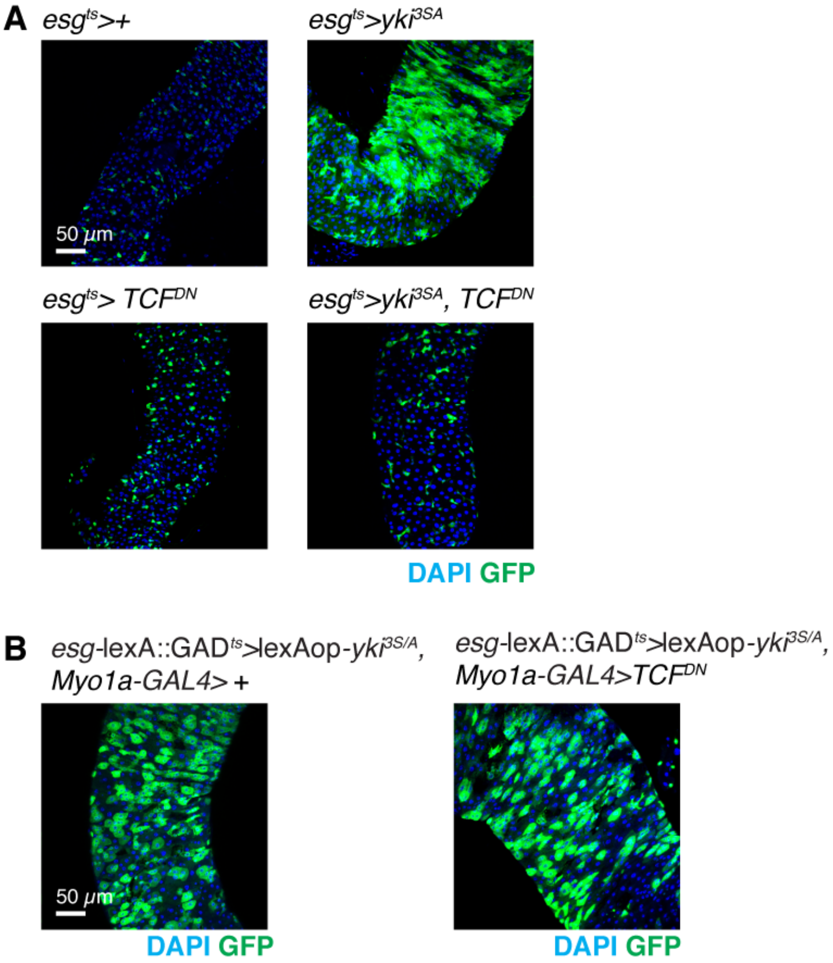

Figure S3. Wg signaling in *yki*$^{3S/A}$ tumor cells is required for *yki*$^{3S/A}$ tumor growth.

(A) Representative images of posterior midguts. Transgenes were expressed for 5 days with *esg*^{ts}. Scale bars, 50 µm.

(B) Representative images of posterior midguts. Expression of the dominant-negative dTCF (TCF^{DN}) in the neighboring ECs didn't alter the growth of *yki*$^{3S/A}$ tumors. Transgenes were expressed for 5 days with *esg*^{ts}. Scale bars, 50 µm.

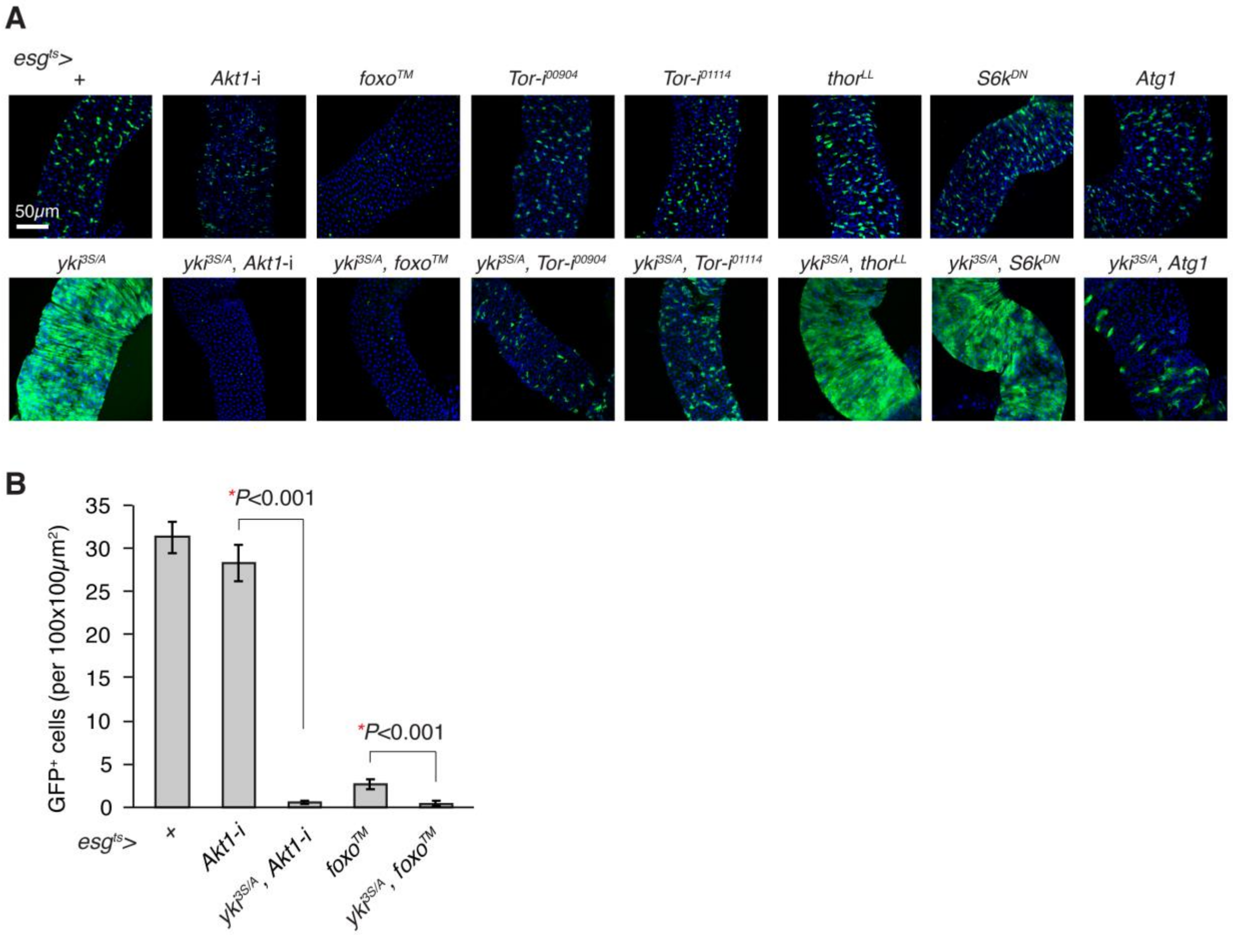

Figure S4. Activation of Foxo or Atg1 attenuates ***yki***$^{3S/A}$ **tumor growth.**

(A) Representative images of posterior midguts. Transgenes were induced for 5 days with *esg*^{ts}. GFP (green) marks *esg*$^{+}$ cells, and nuclei are stained with DAPI (blue). RNAi lines: *Akt-i*, *HMS00007*; *Tor-i*^{00904}, *HMS00904*; *Tor-i*^{01114}, *HMS01114*. Scale bar, 50 µm.

(B) Quantification of GFP^{+} cell number. The number of *esg*$^{+}$ cells in a representative 100 µmx100 µm area in each posterior midgut were quantified. N=10 (*esg*^{ts}>+), N=11 (*esg*^{ts}>*Akt-i*), N=10 (*esg*^{ts}>*yki*$^{3S/A}$, *Akt-i*), N=10 (*esg*^{ts}>*foxo*^{TM}), N=11 (*esg*^{ts}>*yki*$^{3S/A}$, *foxo*^{TM}). Mean±SEMs are shown. *P<0.01, two-tailed unpaired Student's *t*-test between two genotypes indicated by bracket.

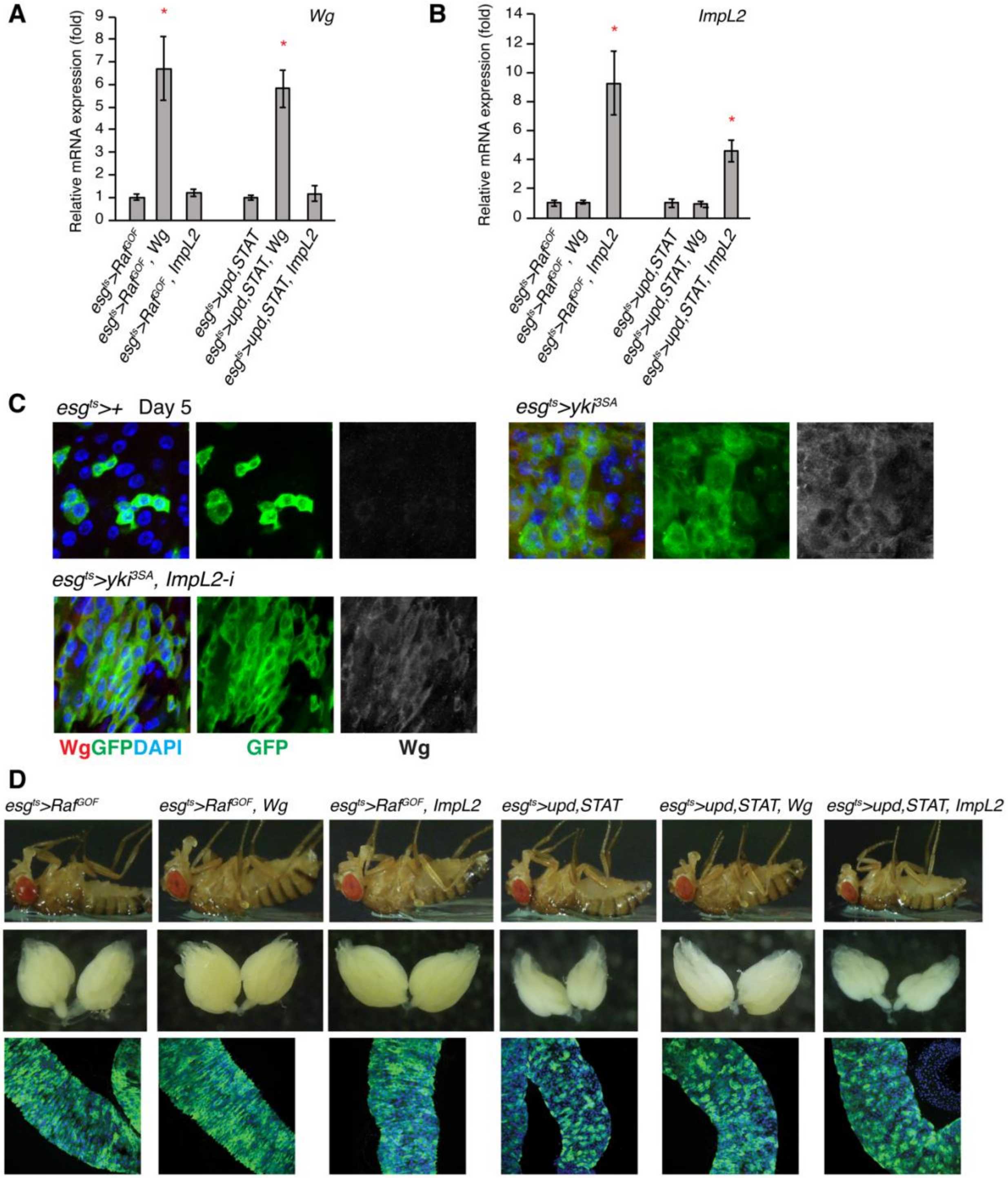
A
Wg
Relative mRNA expression (fold)
9
8
7
6
5
4
3
2
1
0
*
*
esgts>RafGOF
esgts>RafGOF, Wg
esgts>RafGOF, ImpL2
esgts>upd,STAT
esgts>upd,STAT, Wg
esgts>upd,STAT, ImpL2
B
ImpL2
Relative mRNA expression (fold)
14
12
10
8
6
4
2
0
*
*
esgts>RafGOF
esgts>RafGOF, Wg
esgts>RafGOF, ImpL2
esgts>upd,STAT
esgts>upd,STAT, Wg
esgts>upd,STAT, ImpL2
C
esgts>+ Day 5
esgts>yki3SA
esgts>yki3SA, ImpL2-i
WgGFPDAPI
GFP
Wg
D
esgts>RafGOF
esgts>RafGOF, Wg
esgts>RafGOF, ImpL2
esgts>upd,STAT
esgts>upd,STAT, Wg
esgts>upd,STAT, ImpL2

Figure S5. Wg overexpression not sufficient to induce ImpL2 expression, and ImpL2 is not the key regulator of Wg expression.

(A, B) Quantification of *wg* and *ImpL2* transcripts. *UAS-Wg* and *UAS-ImpL2* were expressed with esg^{ts} *driver in* Raf^{gof} and *upd1, Stat92E* tumors. qRT-PCR was performed after 5 days of transgene expression. Mean±SEMs are shown. *P<0.01, two-tailed unpaired Student's t-test compared with control (esg^{ts}> $\mathrm{Raf}^{\mathrm{gof}}$ or esg^{ts}> *upd1, Stat92E*).

(C) Immunostaining of Wg. Transgenes were expressed for 5 days with esg^{ts}.

(D) Representative images of fly, ovary, and posterior midgut.

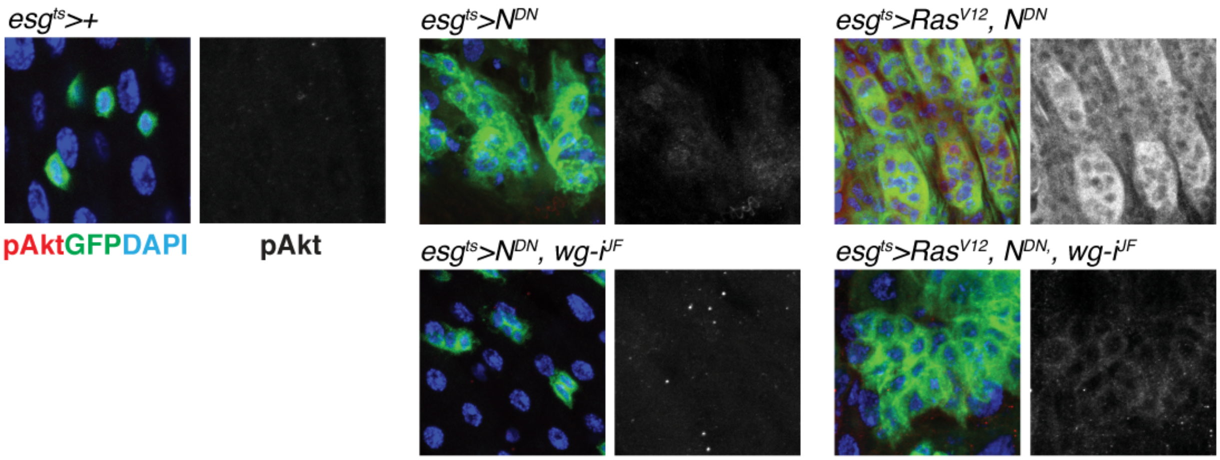

Figure S6. Wg plays a critical role in increasing Insulin signaling in tumors with elevated ImpL2 levels.

Phospho-Akt (pAkt) immunostaining in the midguts. Transgenes were expressed for 5 days. Insulin signaling is upregulated in *esg*^{ts}>*N*^{DN} and *esg*^{ts}>*Ras*^{V12}, *N*^{DN} midguts and reduced by *wg* depletion.

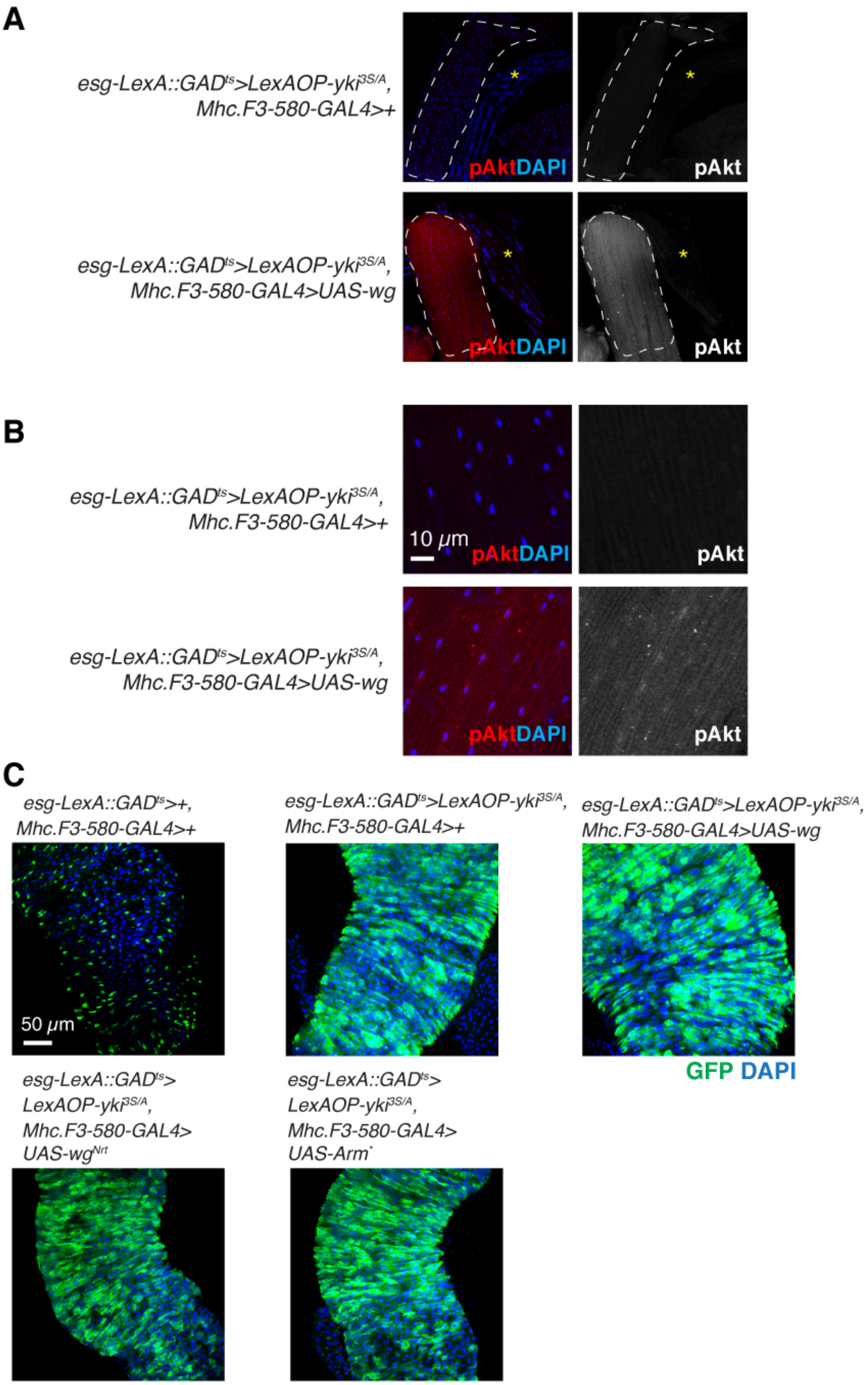
A
esg-LexA::GAD^{ts}>LexAOP-yki$^{3S/A}$,
Mhc.F3-580-GAL4>+
esg-LexA::GAD^{ts}>LexAOP-yki$^{3S/A}$,
Mhc.F3-580-GAL4>UAS-wg
pAktDAPI
pAkt
B
esg-LexA::GAD^{ts}>LexAOP-yki$^{3S/A}$,
Mhc.F3-580-GAL4>+
esg-LexA::GAD^{ts}>LexAOP-yki$^{3S/A}$,
Mhc.F3-580-GAL4>UAS-wg
10 μm
pAktDAPI
pAkt
C
esg-LexA::GAD^{ts}>+,
Mhc.F3-580-GAL4>+
esg-LexA::GAD^{ts}>LexAOP-yki$^{3S/A}$,
Mhc.F3-580-GAL4>+
esg-LexA::GAD^{ts}>LexAOP-yki$^{3S/A}$,
Mhc.F3-580-GAL4>UAS-wg
50 μm
GFP DAPI
esg-LexA::GAD^{ts}>
LexAOP-yki$^{3S/A}$,
Mhc.F3-580-GAL4>
UAS-wg^{Nrt}
esg-LexA::GAD^{ts}>
LexAOP-yki$^{3S/A}$,
Mhc.F3-580-GAL4>
UAS-Arm*

Figure S7. Expression of Wg with *Mhc.F3-580-GAL4* increases Akt phosphorylation in the indirect flight muscle but has no effect on the growth of *yki*$^{3S/A}$ tumors in the midgut.

(A) *esg-LexA::GAD*^{ts}*/Mhc.F3-580-GAL4; LexAOP-yki*$^{3S/A}$*/wg* thorax shows elevated phospho-Akt staining in the indirect flight muscle (white dotted line), but not in the neighboring muscle compartment (asterisks). Phospho-Akt staining is shown in red, and nuclei are stained with DAPI (blue).

(B) Elevated pAkt levels in the thorax confirmed in female.

(C) Representative images of posterior midguts. Transgenes were induced for 6 days. GFP (green) marks *esg*$^{+}$ cells, and nuclei are stained with DAPI (blue). Scale bar, 50 µm.

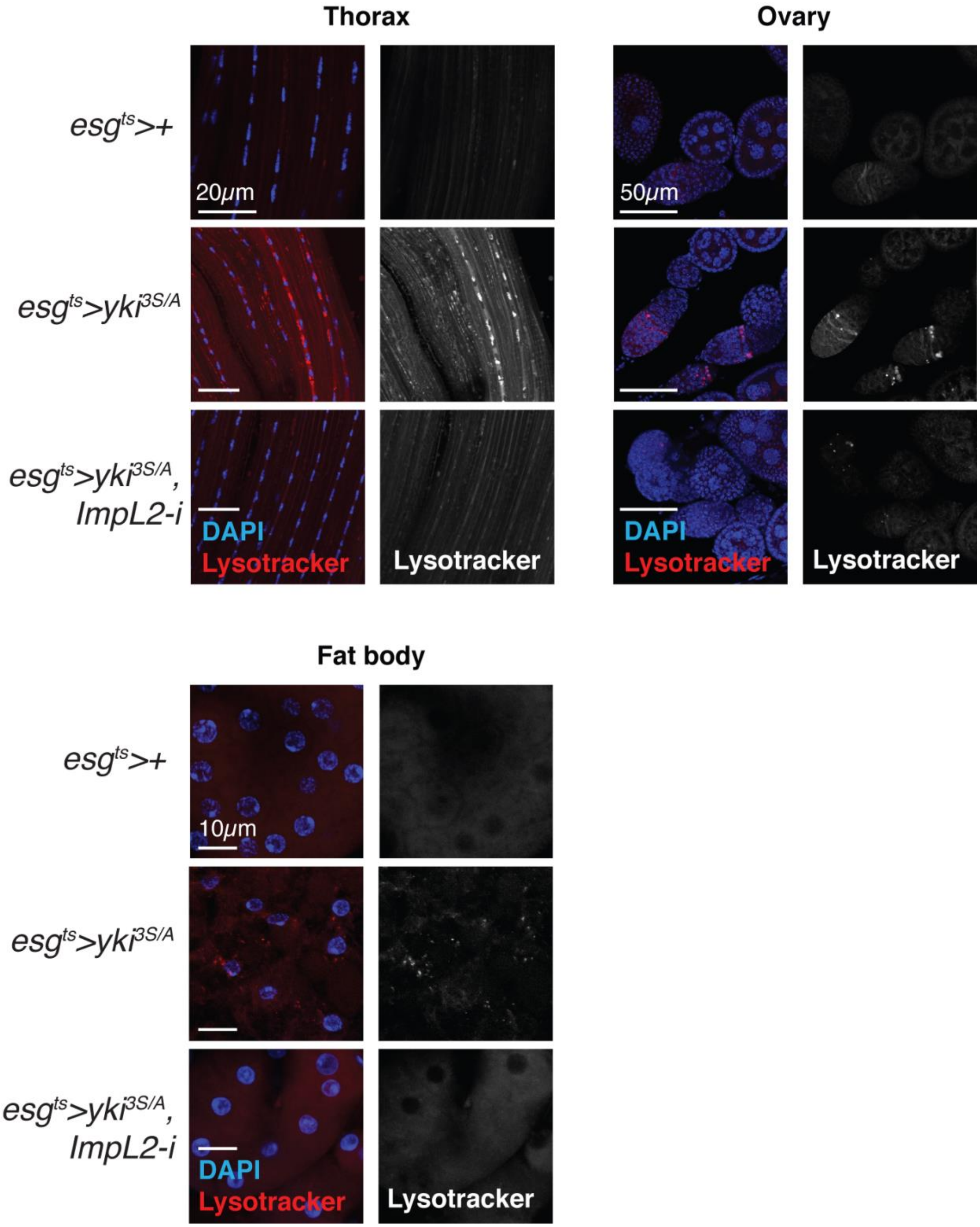
Thorax
Ovary
esgts>+
esgts>yki3S/A
esgts>yki3S/A,
ImpL2-i
20μm
50μm
DAPI
Lysotracker
Lysotracker
DAPI
Lysotracker
Lysotracker
Fat body
esgts>+
esgts>yki3S/A
esgts>yki3S/A,
ImpL2-i
10μm
DAPI
Lysotracker
Lysotracker

Figure S8. Depletion of *ImpL2* in *yki*$^{3S/A}$ tumors is sufficient to suppress autophagy in the host tissues.

Lysotracker staining in thorax, ovary, and fat body. Transgenes were expressed for 5 days. Tissues were stained with LysoTracker Red DND99 (red in merge), and counterstained for nuclei with DAPI (Blue). Scale bars are as indicated.

References

1. Y. Kwon *et al.*, Systemic organ wasting induced by localized expression of the secreted insulin/IGF antagonist ImpL2. *Dev Cell* **33**, 36-46 (2015).
